# Growth resistance and resilience of mixed silver fir and Norway spruce forests in central Europe: Contrasting responses to mild and severe droughts

**DOI:** 10.1111/gcb.15737

**Published:** 2021-06-24

**Authors:** Alessandra Bottero, David I. Forrester, Maxime Cailleret, Ulrich Kohnle, Arthur Gessler, Dominic Michel, Arun K. Bose, Jürgen Bauhus, Harald Bugmann, Matthias Cuntz, Loïc Gillerot, Marc Hanewinkel, Mathieu Lévesque, James Ryder, Julien Sainte‐Marie, Julia Schwarz, Rasoul Yousefpour, Juan Carlos Zamora‐Pereira, Andreas Rigling

**Affiliations:** ^1^ Swiss Federal Institute for Forest, Snow and Landscape Research WSL Birmensdorf Switzerland; ^2^ SwissForestLab Birmensdorf Switzerland; ^3^ Chair of Silviculture Faculty of Environment and Natural Resources University of Freiburg Freiburg Germany; ^4^ UMR RECOVER Aix Marseille University INRAE Aix‐en‐Provence France; ^5^ Forest Research Institute of Baden‐Württemberg FVA Freiburg Germany; ^6^ Institute of Terrestrial Ecology ETH Zürich Zürich Switzerland; ^7^ IT Services Group Department of Health Sciences and Technology ETH Zürich Zürich Switzerland; ^8^ Forest Ecology Department of Environmental Systems Science ETH Zürich Zürich Switzerland; ^9^ Forest and Wood Technology Discipline Khulna University Khulna Bangladesh; ^10^ Université de Lorraine AgroParisTech INRAE UMR Silva Nancy France; ^11^ Forest Management & Silviculture Department of Environmental Systems Science ETH Zürich Zürich Switzerland; ^12^ Chair of Forestry Economics and Forest Planning University of Freiburg Freiburg Germany

**Keywords:** *Abies alba*, adaptation strategies, climate change, forest management, inventory data, *Picea abies*, species interaction, tree rings

## Abstract

Extreme droughts are expected to increase in frequency and severity in many regions of the world, threatening multiple ecosystem services provided by forests. Effective strategies to adapt forests to such droughts require comprehensive information on the effects and importance of the factors influencing forest resistance and resilience. We used a unique combination of inventory and dendrochronological data from a long‐term (>30 years) silvicultural experiment in mixed silver fir and Norway spruce mountain forests along a temperature and precipitation gradient in southwestern Germany. We aimed at examining the mechanisms and forest stand characteristics underpinning the resistance and resilience to past mild and severe droughts. We found that (i) fir benefited from mild droughts and showed higher resistance (i.e., lower growth loss during drought) and resilience (i.e., faster return to pre‐drought growth levels) than spruce to all droughts; (ii) species identity determined mild drought responses while species interactions and management‐related factors strongly influenced the responses to severe droughts; (iii) intraspecific and interspecific interactions had contrasting effects on the two species, with spruce being less resistant to severe droughts when exposed to interaction with fir and beech; (iv) higher values of residual stand basal area following thinning were associated with lower resistance and resilience to severe droughts; and (v) larger trees were resilient to mild drought events but highly vulnerable to severe droughts. Our study provides an analytical approach for examining the effects of different factors on individual tree‐ and stand‐level drought response. The forests investigated here were to a certain extent resilient to mild droughts, and even benefited from such conditions, but were strongly affected by severe droughts. Lastly, negative effects of severe droughts can be reduced through modifying species composition, tree size distribution and stand density in mixed silver fir‐Norway spruce forests.

## Graphical abstract

We examined mechanisms and stand characteristics underpinning the growth resistance (Rt), recovery (Rc) and resilience (Rs) to past mild and severe droughts in mixed silver fir and Norway spruce mountain forests in southwestern Germany. We found that the forests investigated were to a certain extent resilient to mild droughts, and even benefited from such conditions, but were strongly affected by severe droughts (especially those stands with higher residual basal area and larger trees). Lastly, negative effects of severe droughts can be reduced through modifying species composition, tree size distribution and stand density in mixed silver fir‐Norway spruce forests.

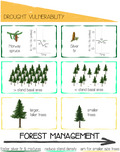

## 1 INTRODUCTION

Gradual changes in climate and the increasing occurrence of extreme climatic events and disturbances are threatening the ecological stability of forests as well as multiple ecosystem services they provide (McDowell et al., 2018; Seidl et al., 2017). The stability properties of a system can be viewed as being composed of resistance, that is, the degree to which the system state changes following a perturbation, and resilience, that is, the processes that allow the system to return to its pre‐perturbation state (Larsen, 1995; Pimm, 1984). These stability concepts can be applied at different levels, for example, to tree populations or to individual trees (Lloret et al., 2011; Schwarz et al., 2020). Maintaining or improving resistance and resilience is an important goal of sustainable forest management to stabilize forest functions and assure the continuous provisioning of ecosystem services in the face of climate change (Scheffer et al., 2001; Seidl et al., 2016).

Among extreme climatic events, droughts are projected to increase in frequency and severity in many regions of the world (IPCC, 2014), with detrimental impacts on the resistance and resilience of individual trees and forest ecosystems in all climatic regions in which forests occur (Bauhus, Forrester, Gardiner, et al., 2017; McDowell et al., 2018). Drought can affect the carbon and water balance, impair plant functioning, reduce primary and secondary growth, impede tree recruitment and increase tree mortality (Anderegg et al., 2013; Schuldt et al., 2020; Senf et al., 2020). The effects of drought on plant functioning may thus trigger large changes in productivity, structure, composition and distribution of entire forest ecosystems (Ciais et al., 2005; Rigling et al., 2013).

Biodiversity is often considered a key feature supporting the resistance and resilience of ecosystem functions to extreme droughts (Isbell et al., 2015; Oliver et al., 2015). Owing to the complementarity among tree species, forest biodiversity may contribute to climate change mitigation by enhancing and stabilizing the productivity of mixed‐species forests at stand to biome scales (del Río et al., 2017; Jucker et al., 2014). Biodiversity–ecosystem functioning relationships in forests are, however, not always positive and vary considerably across regions (Grossiord, Granier, Ratcliffe, et al., 2014), showing a strong dependence on the environmental context (Ratcliffe et al., 2017) and the actual species composition (Ammer, 2019). This applies also to the resistance and resilience to stress, including drought (Bauhus, Forrester, Gardiner, et al., 2017; Forrester et al., 2016; Grossiord, 2020). A positive effect of species diversity on growth resistance and/or resilience to drought of individual tree species has been observed for European beech (*Fagus sylvatica* L.; Pretzsch et al., 2013) and silver fir (*Abies alba* Mill.; Gazol & Camarero, 2016; Vitali, Forrester, et al., 2018), whereas the opposite effect has been documented for Norway spruce (*Picea abies* (L.) Karst.; Vitali, Forrester, et al., 2018). Only weak interspecific effects were found for silver fir and Norway spruce (Dănescu et al., 2018).

In addition to regulating species composition, forest management can have a profound effect on competition for water and light via changing stand density and tree size distribution. Water is a prime limiting factor for plant functioning in drought‐prone environments particularly during hot or dry periods, whereas competition for light is more important on sites generally not limited by water (Forrester, 2014). However, few studies have quantified both water and light competition along environmental gradients for a given species (Forrester, 2014). Maintaining stands at reduced levels of basal area to avoid strong competition is advocated as a management strategy to impart resistance and/or resilience to drought (e.g., Bottero et al., 2017; Sohn et al., 2016), with potential positive repercussions on the adaptation and/or mitigation potential of forest ecosystems (Brang et al., 2014). Especially under generally dry conditions, stand basal area reductions were found to increase water availability and reduce drought stress of the remaining trees (Giuggiola et al., 2016; Manrique‐Alba et al., 2020). This effect, however, may vary among sites (cf. Simon et al., 2017) and depend on drought severity (Gleason et al., 2017).

In the face of climate change, concerns have been raised about the future performance of economically important tree species and the sustainability of the valuable ecosystem services provided by European forests (Hanewinkel et al., 2013). Silver fir and Norway spruce are two of the most abundant and economically important tree species in Europe (San‐Miguel‐Ayanz et al., 2016), whose distribution has been profoundly affected by millennia of human interventions (Caudullo et al., 2016; Tinner et al., 2013). Potential range shift and dynamic vegetation models indicate that species like Norway spruce are likely to decline in the absence of adaptation measures, with severe economic (Hanewinkel et al., 2013) and ecological repercussions (Mina et al., 2017; Temperli et al., 2012). Silver fir may also be negatively affected by increasing aridity mostly at its south‐western distribution limit but may, in contrast, respond positively to warming outside Mediterranean areas, at high elevation or otherwise cool sites, where its growth is currently temperature‐limited (Gazol et al., 2015; Vitasse, Bottero, Rebetez, et al., 2019). Some of these changes in tree species composition have recently accelerated through the widespread tree mortality triggered by the extreme dry and hot years in 2018 and 2019, and associated with extensive bark beetle outbreaks (cf. Schuldt et al., 2020). There is, hence, a strong interest in developing forest management adaptation strategies based on robust information about the effects and importance of different factors influencing tree resistance and resilience, such as climate, management, species interactions and mechanisms that regulate responses to stress, including drought (Yousefpour et al., 2017).

Numerous dendroecological studies on silver fir and Norway spruce support the higher growth susceptibility of Norway spruce to drought (e.g., Lévesque et al., 2013; Vitasse, Bottero, Cailleret, et al., 2019). Most of these studies, however, focus on dominant and/or codominant trees, or ignore the effects of tree size on the drought response, potentially leading to a large bias of growth rates and trends (Forrester, 2019; Nehrbass‐Ahles et al., 2014) and an overestimation of climate change impacts on forest growth (Klesse et al., 2018). For example, drought may affect different tree size classes to different degrees. However, the evidence for such size effects in uneven‐aged forests is ambiguous (Dănescu et al., 2018; Pretzsch et al., 2018). In addition, many studies have focused on the drought response at the tree level, with no or limited consideration of forest stands, management and other factors (e.g., species interactions and traits related to resource‐use efficiency regarding photosynthetically active radiation or soil resources) that may profoundly affect such responses. Ignoring these effects could also lead to overestimates of the importance of some variables at the expense of others.

Here, we provide an analytical approach to evaluate the drought response at the tree and stand scale that takes advantage of a unique combination of forest inventory and dendrochronological data from a long‐term silvicultural experiment to examine the mechanisms and stand characteristics underpinning the growth resistance and resilience to drought of silver fir and Norway spruce growing in mixed stands along a temperature and precipitation gradient in southwestern Germany. The analyzed stands are part of a research project initiated between 1979 and 1981 (shelterwood experiment; Weise, 1995) and were subjected to different experimental thinning intensities leading to different levels of stand densities.

The three specific objectives of this study were as follows.

1. To evaluate the tree‐ and stand‐level reactions of silver fir and Norway spruce growing in mixtures to past drought events of different severity; we hypothesized that each species would show different responses to mild and severe drought events, with silver fir being more resistant and resilient to drought than Norway spruce. Additionally, we hypothesized that stand‐level responses would be less pronounced than those at the individual tree level.
2. To quantify the relative importance of different tree‐, site‐ and drought‐related factors that influence tree‐ and stand‐level growth responses to drought, we hypothesized that different factor combinations would influence tree‐ and stand‐level responses to mild vs. severe droughts, and that the effect of management would be weaker than what was observed in drier environments.
3. Lastly, to provide quantitative information on stand characteristics and tree growth‐related mechanisms that support the drought resistance and resilience of the two species to advise forest management in the face of climate change.

## 2 MATERIALS AND METHODS

### 2.1 Study sites

**FIGURE 1:**
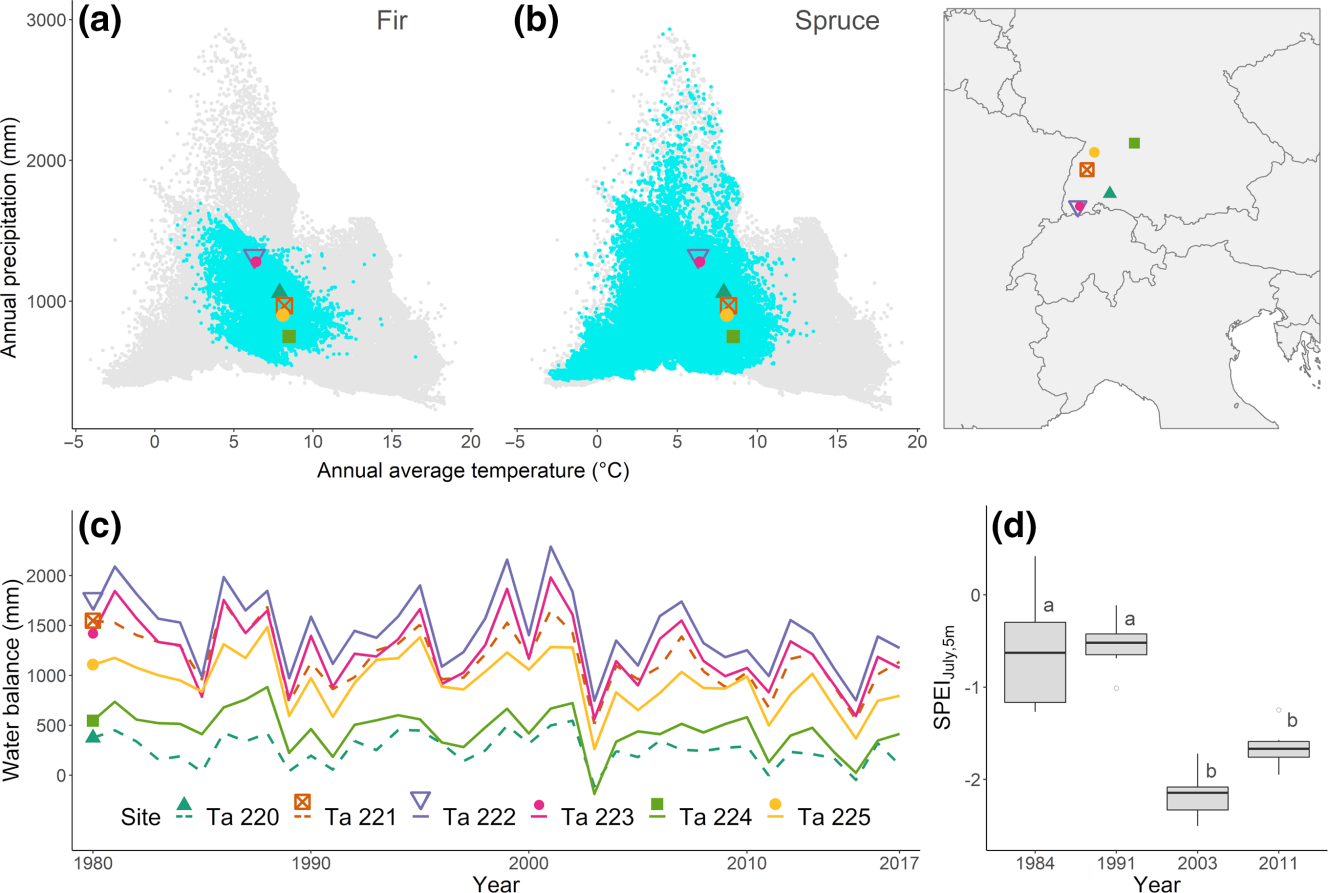
Location of study sites in central Europe, with climate‐space diagrams for (a) fir and (b) spruce; gray dots are all forest field observations in Europe, cyan dots are observed presence of the species in Europe and different symbols denote the six study sites. Presence data and temperature and precipitation data used in the climate‐space diagrams were extracted from the European Atlas of Forest Tree Species (San‐Miguel‐Ayanz et al., 2016), and from WorldClim 2 (Fick & Hijmans, 2017) for the study sites. (c) Annual climatic water balance (annual sum of precipitation – annual potential evapotranspiration) for the period 1980–2016 across study sites. (d) Box plots of the Standardized Precipitation and Evapotranspiration Index of July at the time scale of 5 months (SPEI_July,5m_) for the four drought events analyzed. Different letters indicate significant differences among years (ANOVA test, *α* < 0.05)

**FIGURE 2:**
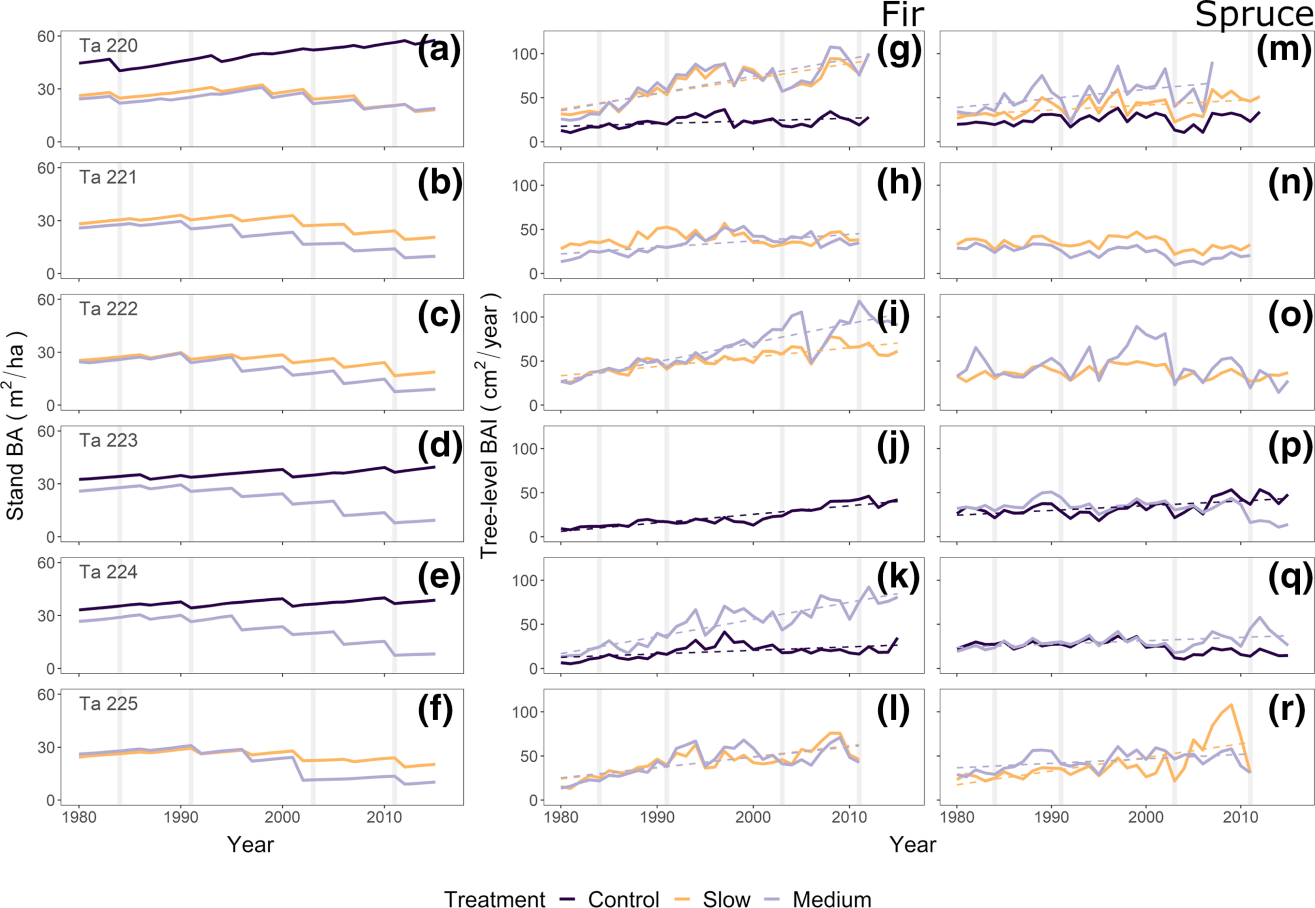
Stand basal area (BA, m^2^/ha, panels a–f) and mean annual tree‐level basal area increment (BAI, cm^2^/year) of fir (panels g–l) and spruce (panels m–r) across the analyzed treatments and sites (Ta 220–225) since 1980 (see Figure S9 for tree‐ring width indices). Linear regression lines (dotted) in panels (g–r) show significant growth trends. Vertical gray lines denote the years 1984, 1991, 2003 and 2011

The six study sites are located in southwestern Germany and distributed over the major natural range of silver fir and Norway spruce in the Black Forest (Schwarzwald), the Swabian‐Franconian Forest (Schwäbisch‐Fränkischer Wald) and the southwestern Swabian Jura (Schwäbische Alb; Weise, 1995; Figure 1). They support mixed‐species mountain forests consisting mainly of silver fir (*Abies alba* Mill.; hereafter called fir), Norway spruce (*Picea abies* (L.) Karst.; hereafter called spruce) and European beech (*Fagus sylvatica* L.; hereafter called beech), with interspersed individuals of Scots pine (*Pinus sylvestris* L.). The climate is temperate to cool‐temperate and mean total annual precipitation ranges from c. 910 to 1920 mm for the period 1881–2016 (Table 1). The warmest month is July (15.1 ± 1.9℃) and the coldest month is January (−1.8 ± 2.6℃) at all sites. The study sites are part of a long‐term silvicultural experiment initiated between 1979 and 1981 to investigate tree and regeneration responses to the irregular group shelterwood regeneration method (*Femelschlag*; Puettmann et al., 2009; Weise, 1995). Before the initiation of the experiment, the stands were mature even‐aged, naturally regenerated and no harvesting interventions had taken place in the decade preceding the installation of the plots. At the time of the research installation, the treatments were cut to 75% of the volume of a fully stocked stand. The experimental stands analyzed in this study comprised two *Femelschlag* regeneration cutting regimes (treatments) differing in terms of the length of the regeneration period (and, thus, the speed of removal of canopy trees, namely slow and medium) and increment controls (stands maintained fully stocked via the periodic removal mostly of dead and damaged trees). The interventions were planned at 5‐year intervals in each stand (for more details on the experiment, see Appendix S1). This resulted in a variety of tree growth conditions reflecting a range of stand basal area (Figure 2a–f), tree stem density (Figure S1), vertical structure (Dănescu et al., 2016) and different levels of intraspecific and interspecific competition (Forrester et al., 2013).

**TABLE 1:** Site and stand information of the six study sites in southwestern Germany

	Ta 220	Ta 221	Ta 222	Ta 223	Ta 224	Ta 225
Region	Swabian Jura	Black Forest	Black Forest	Black Forest	Swabian‐Franconian Forest	Black Forest
Latitude	47°58,4′	48°25,8′	47°43,9′	47°44,0′	48°56,2′	48°45,7′
Longitude	8°52,5′	8°13,9′	7°58,4′	8°1,5′	9°34,0′	8°26,4′
Elevation (m a.s.l.)	830	720	1020	1020	520	700
Slope (°)	3	26	14	12	7	14
Aspect (°)	53	127	170	75	184	63
Parent material^a^	Limestone	Sandstone	Gneiss	Granite	Sandstone	Sandstone
Soil conditions^a^	Well drained, loamy	Well drained, sandy	Well drained, loamy	Well drained, loamy	Sandy top layer over loamy layer	Well drained, sandy, acid
MAT (°C) 1881–1979/1980–2016	6.8/7.4	6.4/7.2	4.8/5.5	5.3/6.0	7.6/8.3	6.8/7.5
MAP (mm) 1881–1979/1980–2016	900/947	1679/1758	1887/2019	1703/1849	1044/1149	1441/1550
Mean basal area (m^2^/ha) 1979	47.9	42.1	41.0	44.2	39.5	39.6
Mean tree age^a^ fir/spruce (years) 1979	103/91	120/126	95/92	116/107	108/103	98/91
Mean tree height^a^ fir/spruce (m) 1979	28.9/29.9	25.8/27.1	29.8/30.9	29.8/30.9	29.7/30.4	27.4/29.0
Proportion^a^ fir/spruce (%) 1979	76/20	43/57	60/24	23/63	37/63	53/47
Number of stands analyzed	5	2	2	2	3	2
Number of trees inventoried	380	214	199	329	345	207
Number of discs collected	81	51	20	31	75	31

Abbreviations: MAP, mean total annual precipitation; MAT, mean annual temperature.

^a^ Weise (1995).

This experiment was chosen for our study because (i) the plots are part of a well‐documented experiment lasting >30 years, which is based on a strict experimental design and measurement protocol; (ii) the analyzed growth responses to drought are based on trees living at the time of sampling and on those progressively harvested over time, in contrast to most dendroecological studies using only living trees at the time of sampling, thereby missing the responses of trees that had previously been harvested or died; (iii) mortality events were precisely recorded at the tree level, thus eliminating uncertainties related to unknown past mortality (Teets et al., 2018); (iv) detailed records of individual‐tree positions and dimensions, including height, crown length and crown radii, were measured repeatedly, therefore allowing also for an estimation of tree–tree competition at the annual level over time; and (v) it was possible to collect additional tree‐ring samples from some of the fully stocked control stands within the experiment, which is often not allowed in managed stands or long‐term experiments.

### 2.2 Inventory and field data collection, and laboratory analysis

At the onset of the long‐term experiment in 1979, on the respective treatment or control plots (plot area c. 0.25 ha), all trees ≥4.0 cm in diameter at breast height (DBH, 1.3 m height) were mapped, measured (DBH, height, crown length and radii) and tagged, recording c. 2000 trees in total. All tagged living trees were periodically remeasured at c. 5‐year intervals, at the end of the growing season. Tree removal occurred during the winter, before the beginning of the following growing season. Trees of poor quality or health, or that had died or disappeared during the interval between inventories were also recorded. The cause and time of death were assessed and the DBH and height at the time of death reconstructed. Annual tree radial growth was measured on discs collected at breast height from 289 trees harvested progressively between 1980 and 2017 from the treatment and control plots (Table 1). Cross‐sections were air‐dried, sanded with progressively finer sandpaper and visually cross‐dated. Radial increments were measured using a Digitalpositiometer Typ 2 (developed by K. Johann; Biritz GmbH, precision 0.01 mm), and TSAPWin^TM^ (Rinntech, 2003) using the Lintab measuring device (precision 0.01 mm). In total, 2012 individual measurement series were obtained (eight radii per disc for trees measured at the Forest Research Institute of Baden‐Württemberg FVA, three radii per disc for trees measured at the Swiss Federal Institute for Forest, Snow and Landscape Research WSL) across the six sites. Cross‐dating and ring‐width measurements of each series were checked for errors using the COFECHA software (Holmes, 1983). Ring series were converted to annual tree basal area increment (BAI) based on backwards‐reconstructed DBH values derived from DBH inside bark at time of sampling and radial increments over time (Bunn, 2008). The harvested trees were used to develop species‐specific bark factor equations for bark thickness as a function of DBH, then validated against regional bark thickness models (Stängle & Dormann, 2018; Stängle et al., 2016). Bark thickness was subtracted from DBH to obtain the corresponding DBH inside bark. BAI was used instead of ring width because of its lower dependence on stem size and cambial age (Biondi, 1999), and because BAI is more representative of biomass increment (Bouriaud et al., 2005). Stand‐level BAI was quantified as the sum of tree‐level BAI for each stand and year. To obtain annual data for all trees without tree‐ring width series, DBH, height, crown length, crown projections and leaf area were reconstructed using linear and nonlinear least square regression models (for details on the methods see Appendix S1).

### 2.3 Climate data and drought variables

Downscaled monthly air temperature (minimum, mean and maximum) and precipitation sum for each study site were obtained from the DWD Climate Data Center dataset (1 × 1 km grids over Germany, version v1.0; Kaspar et al., 2013). Based on these variables, the multiscalar Standardized Precipitation and Evapotranspiration Index (SPEI, unitless; Vicente‐Serrano et al., 2010) was used as an indicator for climatic drought, and calculated with the R package *SPEI* (Beguería & Vicente‐Serrano, 2013). The potential evapotranspiration (PET) used in the function to obtain the series of monthly climatic water balance (water balance = precipitation − PET) was calculated according to the Hargreaves equation (Hargreaves, 1994), and the SPEI was then computed at different time scales. Correlation analyses between SPEI and tree‐ring chronologies showed that the most suitable SPEI time window across all sites was the 5‐month period from March to July (SPEI_July,5m_, Figure S2), which corresponds to the period just before and during which most of the cambial activity occurs for the study species in the region (Dietrich et al., 2018).

Four single‐year climatic drought events, corresponding to radial growth depressions, were selected for analysis. The first two (years 1984 and 1991, hereafter called mild drought events) had the lowest average values of SPEI_July,5m_ across sites throughout the first half of the experiment (SPEI_July,5m_ = −0.61 ± 0.66 in 1984 and −0.54 ± 0.30 in 1991); the other two events (years 2003 and 2011, hereafter called severe drought events) had the lowest average values of SPEI_July,5m_ across sites after 2000 (SPEI_July,5m_ = −2.16 ± 0.27 in 2003 and −1.65 ± 0.24 in 2011, Figure 1d; Figure S3). The year 2014, although severe, was not included in the analysis due to a low number of observations in the inventory data.

### 2.4 Stand structure and composition, tree resource use, and management‐related variables

A wide variety of stand structure‐ and composition‐related variables (e.g., species interaction, species composition), traits related to resource‐use efficiency (absorption of photosynthetically active radiation) and management‐related variables (reflecting the intensity and frequency of thinning and, thus, the experimental treatments and controls) was used as tree‐ and stand‐level predictors of growth responses to drought (Table S1).

At the tree level, a distance‐dependent competition index (NI) was computed:

$${\text{NI}}_{t}=\sum_{i=1}^{n}\frac{{\text{BA}}_{i}}{{\text{distance}}_{\mathrm{ti}}},$$

where NI*_t_* is the competition intensity experienced by tree*_t_*, from *n* neighboring trees*_i_* within a radius of 10 m of tree*_t_*; BA is the tree*_i_* basal area (cm^2^); and distance*_ti_* is the distance (m) between the stem center of the central tree*_t_* and its *i*th neighbor. Along a continuous range of radii, 10 m was selected because it maximized the *R* ^2^ of the relationship between the periodic annual basal area increment and NI*_t_* (Forrester et al., 2013). To account for differences in species interactions among neighboring trees, the proportion of competition intensity (NI*_t_*) exerted by fir, spruce and other species was computed as a ratio of NI*_spX_* to NI*_t_*, where NI*_spX_* is the NI*_t_* calculated only considering fir, spruce or other species as neighboring trees, respectively.

Individual‐tree absorption of photosynthetically active radiation (APAR, GJ/tree/year) was predicted using the 3D tree‐level model Maestra (Medlyn, 2004; Wang & Jarvis, 1990). Maestra APAR predictions have been tested and validated in several monospecific and mixed forests (Forrester et al., 2018; Wang & Jarvis, 1990). Crown architecture (live crown length and radius, leaf area) and species‐specific differences in leaf optical properties, as well as leaf area density and angle distributions were used to predict individual‐tree APAR (Table S2). The model defines tree crown positions by *x* and *y* coordinates, considering slope and aspect in both *x* and *y* directions, to account for the shading of neighboring trees in the canopy. Individual tree APAR was computed as total annual APAR for evergreen species, or total APAR of the period between leaf unfolding and leaf discoloration in autumn for deciduous species (Table S2). To avoid edge effects, APAR predictions were not used for trees within 10 m of plot boundaries. An additional 20 m wide buffer was simulated around each plot, and contained stands with the average tree spacing, species composition and tree sizes of the given plot (Forrester & Albrecht, 2014).

At the stand level, the Shannon diversity index (Shannon, 1948) was used as a measure of compositional diversity and was calculated with the R package *vegan* (Oksanen et al., 2013). The relative contribution of fir, spruce and other species to total stand basal area was also considered. The effect of management on drought responses was tested based on variables related to the intensity and frequency of thinning, such as total residual stand basal area at the time of drought (as an expression of the treatment intensity), basal area removed, total number of interventions and the number of years since the last thinning.

### 2.5 Growth responses to drought

Tree‐ and stand‐level growth responses to drought were expressed as resistance, recovery and resilience (Lloret et al., 2011; Figures S4 and S5). These three indices allow for the examination of growth performance before, during and after periods of stress, and therefore characterize tree‐ and stand‐level growth responses to drought. The indices were calculated as follows:

$$\text{Resistance}=\frac{{\text{BAI}}_{\text{Dr}}}{{\text{BAI}}_{\text{preDr}}};\;\text{Recovery}=\frac{{\text{BAI}}_{\text{postDr}}}{{\text{BAI}}_{\text{Dr}}};\;\text{Resilience}=\frac{{\text{BAI}}_{\text{postDr}}}{{\text{BAI}}_{\text{preDr}}},$$

where BAI_Dr_ is the BAI during drought, BAI_preDr_ is the average BAI for the 2 years preceding drought and BAI_postDr_ is the average BAI for the 2 years following drought. The same indices, but calculated using detrended tree‐ring series (smoothing spline with 50% frequency response at 2/3 of series’ length), led to similar results (correlation coefficient *r* > 0.9). A period of 2 years was selected because radial growth autocorrelation >0.5 was found for 1‐ and 2‐year lags (Figure S6), supporting the significant legacies in radial growth observed in non‐arid sites for 1–2 years after drought (Anderegg et al., 2015). Additionally, the short time window pre‐ and post‐drought limits the confounding effect of other biotic and abiotic events that can determine growth anomalies (e.g., the mast year in 2006; Ascoli et al., 2017; Schwarz et al., 2020).

### 2.6 Statistical analyses

Stand structure and composition, traits related to resource‐use efficiency, and management effects on resistance, recovery and resilience to drought were predicted using linear mixed‐effects models (LMM, at the tree level) and linear models (at the stand level). General individual‐tree APAR conditions—average values of APAR of the 2 years preceding drought—were included in the models. First, all weakly correlated predictors (*r* < 0.5, cf. Figure S7 for tree‐ and Figure S8 for stand‐level variables) with their interactions were included as fixed effects in the full model. Additionally, multicollinearity among explanatory variables was assessed using the variance inflation factor (VIF), and variables with VIF > 2 were removed from the full model. A total of six models were fitted at both the tree and the stand level. At each level, three models were developed for each drought group (mild and severe drought events). In the tree‐level models, an interaction with species was added to all predictors. Predictors were scaled and centered to improve interpretability and to allow for the direct comparison of the regression coefficients (Schielzeth, 2010). Different random structures were tested to select the optimal random structure and, thus, type of model for analysis (for details, see Appendix S1; Table S3). Including a random structure in the models with “site” as grouping factor did not improve their performance, highlighting similar drought effects among sites irrespective of their structure, composition and management (Table S3). A set of models with all possible combinations of fixed effect terms in the full model was ranked by the second‐order Akaike information criterion (AIC*c*), and used to select the model with lowest AIC*c* (best model). Marginal and conditional R‐squared and coefficient of determination (Nakagawa & Schielzeth, 2013) were calculated to examine the variance explained by the fixed effects and by the entire model, respectively. The assumptions of normality and variance homogeneity of residuals were visually verified. Estimates of the variance components (effect size) for each predictor variable were obtained as the ratio of the variance of the standardized predictor to the total variance of the best model for each index and drought event. Nonparametric Kruskal–Wallis tests were conducted to detect differences in drought response (resistance, recovery and resilience) among drought events (mild and severe), forest components (individual fir, individual spruce and whole stand) and treatments (control, slow and medium). All analyses were performed in the statistical computing software R (version 4.0.1, R Core Team, 2020) using the packages *lme4* (Bates et al., 2007), *car* (Fox et al., 2012), *MuMIn* (Bartoń, 2015) and *rstatix* (Kassambara, 2020).

## 3 RESULTS

### 3.1 Tree growth

Differences in growth trends were observed for individuals of fir and spruce since 1980. Fir showed significantly increasing growth rates across all treatments and sites (except for the slow treatment at site Ta 221), with differences among treatments (Figure 2g–l). In contrast, spruce showed less pronounced differences among treatments, and several non‐significant growth trends (Figure 2m–r). In accordance with the experimental treatment plans, stand basal area decreased in all harvested stands while it increased in the increment controls (Figure 2a–f).

### 3.2 Influence of drought event, forest component and treatment on drought responses

Drought responses varied considerably between drought events, with significantly lower values of resistance, recovery and resilience observed during severe droughts (Figure S10; Table S4). Compared with individuals of fir and whole stands, individuals of spruce recorded lower resistance, recovery and resilience to mild droughts, but higher recovery to severe droughts; whole stands were significantly more resistant and resilient to severe droughts than the average individual trees of those stands (Figure S10; Tables S4 and S5).

### 3.3 Predictors of tree‐level resistance, recovery and resilience

**FIGURE 3:**
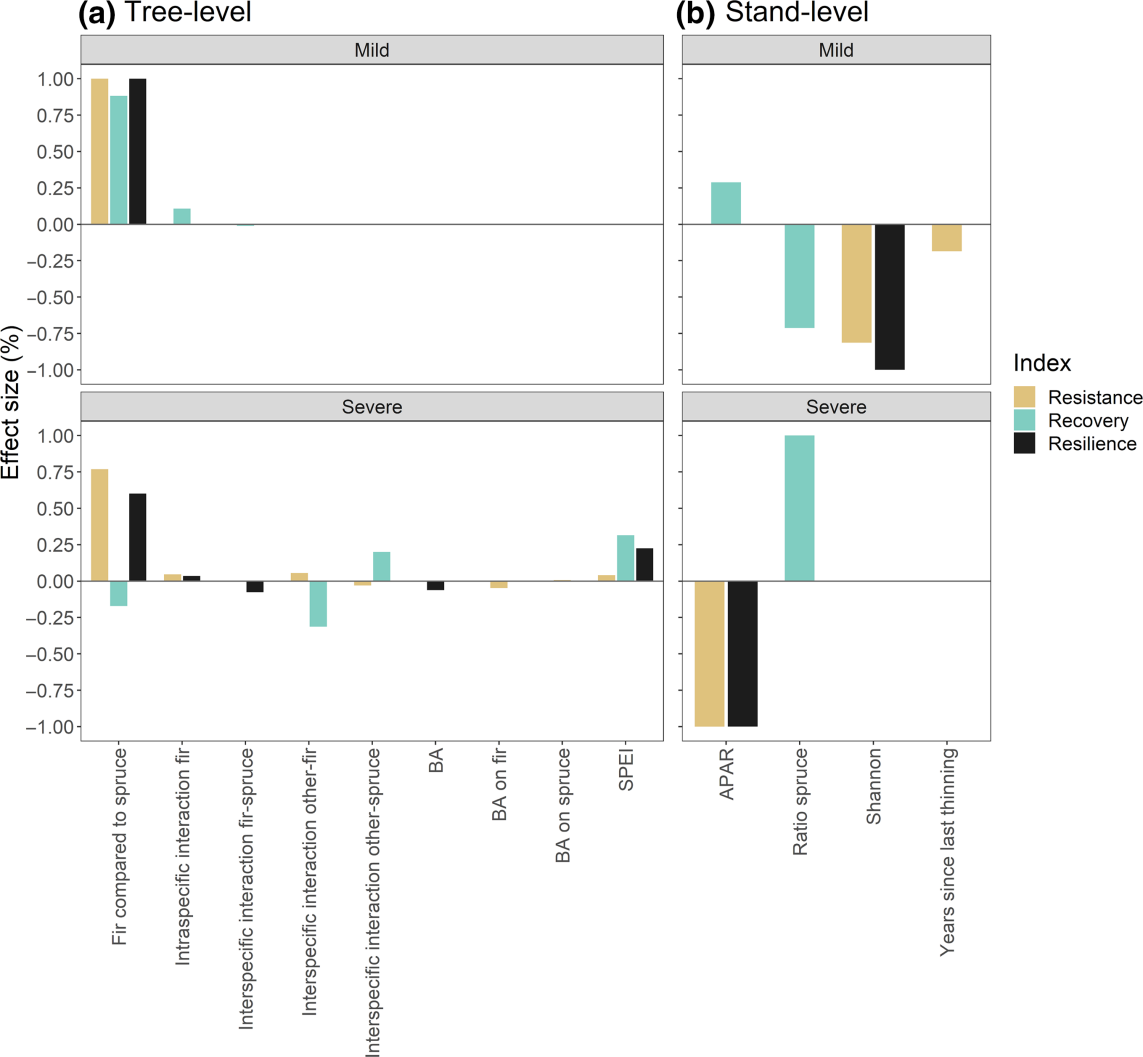
Variance components showing the proportion of total variation in (a) tree‐level and (b) stand‐level resistance, recovery and resilience to mild and severe drought events explained by each predictor variable (effect size). *Fir compared to spruce* = species comparison: response (resistance, recovery and resilience) of fir compared to spruce; *BA* = effect of residual stand basal area on both fir and spruce; *BA on fir/spruce* = effect of residual stand basal area on fir/spruce; *Intraspecific interaction fir* = effect of intraspecific interaction on fir; *Interspecific interaction fir‐spruce* = effect of interspecific interaction of fir with spruce; *Interspecific interaction other‐fir/spruce* = effect of interspecific interaction of other species (mainly beech) with fir/spruce; *SPEI* = effect of SPEI of July at the time scale of 5 months; *APAR* = effect of absorption of photosynthetically active radiation; *Ratio spruce* = effect of ratio of basal area of spruce to total stand basal area; *Shannon* = effect of Shannon diversity index; *Years since last thinning* = effect of the number of years since the last thinning

For mild drought events at the tree level, species identity and species interaction were the main predictors of resistance, recovery and resilience (Table 2; Figure 3a). Fir showed significantly higher resistance, recovery and resilience than spruce, and its recovery benefited from intraspecific interactions (Table 2; Figure 3a). Species identity contributed with 85%–100% to the explained variance across all drought responses (Figure 3a).

**TABLE 2:** Summary of the linear mixed‐effect models

Tree‐level	Best model	Best model	Best model
	Estimate (SE)	Estimate (SE)	Estimate (SE)
Mild	Resistance	Recovery	Resilience
Intercept	−0.47 (0.15)*	−0.41 (0.11)**	−0.55 (0.13)**
Sp_fir_	0.74 (0.10)***	0.61 (0.10)***	0.86 (0.10)***
NI_ratio_fir_		−0.07 (0.09)	
NI_ratio fir_ × Sp_fir_		0.21 (0.10)*	
$R_{m}^{2}$	0.127	0.102	0.170
$R_{c}^{2}$	0.216	0.134	0.240
Severe	Resistance	Recovery	Resilience
Intercept	−0.72 (0.18)**	0.11 (0.17)	−0.56 (0.11)***
Sp_fir_	1.20 (0.12)***	−0.35 (0.14)*	0.88 (0.13)***
NI_ratio_fir_	−0.20 (0.10)*		−0.27 (0.10)**
NI_ratio other_	−0.31 (0.11)**	0.45 (0.12)***	
BA_stand_	0.03 (0.13)		−0.16 (0.07)°
SPEI	0.15 (0.07)*	0.26 (0.08)***	0.27 (0.06)***
NI_ratio fir_ × Sp_fir_	0.30 (0.12)*		0.21 (0.13)
NI_ratio other_ × Sp_fir_	0.32 (0.12)**	−0.46 (0.14)***	
BA_stand_ × Sp_fir_	−0.29 (0.12)*		
$R_{m}^{2}$	0.349	0.172	0.250
$R_{c}^{2}$	0.509	0.265	0.266

Note

Fit of tree‐level resistance, recovery, and resilience (mild and severe events) as a function of different variables (best models). Sp = species (2 levels: fir, and the reference spruce); NI_ratio fir_ = ratio of competition intensity of fir to total intensity of competition; NI_ratio other_ = ratio of competition intensity of other species (mainly beech) to total intensity of competition; BA_stand_ = residual stand basal area; SPEI = SPEI of July at the time scale of 5 months; *x* = interaction; $R_{m}^{2}$ = marginal *R*‐squared (variance explained by the fixed factors); and $R_{c}^{2}$ = conditional *R*‐squared (variance explained by the fixed and random factors). Significance levels: “***” 0.001, “**” 0.01, “*” 0.05, “°” 0.1. See Table S6 for the full models.

For severe drought events, species identity, species interaction, drought severity and residual stand basal area at the time of drought were the main predictors of the analyzed growth responses (Table 2; Figure 3a). Fir showed significantly higher resistance and resilience than spruce, but had lower recovery under severe drought (Table 2). Species identity contributed most to the total proportion of explained variance in resistance and resilience (77% and 60%, respectively) while it had a smaller effect on recovery (17%, Figure 3a). Intraspecific interaction increased the resistance and resilience of both fir and spruce to severe droughts, whereas the resistance and recovery of the two species were differently affected by interspecific interactions (Table 2). Higher proportions of fir in the neighborhood negatively affected the resistance and resilience of spruce to drought while the presence of other species (excluding fir, i.e., mainly beech) negatively affected the resistance of spruce but favored its recovery. The opposite was observed for fir (Table 2), and interspecific interaction (with other species) explained up to 32% of the variance in recovery of fir (Figure 3a). Species interactions had a lower effect in all other cases (<10%, Figure 3a). SPEI exerted a positive effect on all drought responses, particularly recovery and resilience, which were higher under less severe drought conditions (Table 2). Drought severity explained 30% of the variance in recovery and 20% of the variance in resilience (Figure 3a). Higher values of residual stand basal area were associated with lower resistance and resilience (Table 2) and explained <7% of the total variation in the drought response of both species (Figure 3a).

### 3.4 Predictors of stand‐level resistance, recovery and resilience

For mild drought events at the stand level, APAR, proportion of spruce, Shannon diversity index and number of years since the last thinning were the most important predictors of the analyzed growth responses (Table 3). The Shannon diversity index and the number of years since the last thinning both had a negative effect on stand‐level resistance, explaining 81% and 19% of its variance, respectively (Table 3; Figure 3b). Stand‐level recovery was positively influenced by APAR and negatively influenced by the proportion of spruce; APAR and the proportion of spruce explained 29% and 71% of the variance in recovery, respectively (Table 3; Figure 3b). The Shannon diversity index negatively affected the resilience to mild drought events, explaining its entire variance (Table 3; Figure 3b).

**TABLE 3:** Summary of the linear regression models

Stand‐level	Best model	Best model	Best model
	Estimate (SE)	Estimate (SE)	Estimate (SE)
Mild	Resistance	Recovery	Resilience
Intercept	0.00 (0.14)	0.00 (0.17)	0.00 (0.17)
APAR		0.31 (0.18)°	
Shannon	−0.64 (0.15)***		−0.46 (0.18)*
Ratio_spruce_		−0.45 (0.18)*	
Yrs_since last_	−0.30 (0.15)°		
$R_{\text{adj}}^{2}$	0.436	0.192	0.178
Severe	Resistance	Recovery	Resilience
Intercept	0.00 (0.20)	0.00 (0.20)	0.00 (0.20)
APAR	−0.39 (0.20)°		−0.34 (0.21)
Ratio_spruce_		0.38 (0.20)°	
$R_{\text{adj}}^{2}$_j_	0.114	0.107	0.074

Note

Fit of stand‐level resistance, recovery and resilience (mild and severe events) as a function of different variables (best models). APAR = absorption of photosynthetically active radiation; Shannon = Shannon diversity index; Ratio_spruce_ = ratio of basal area of spruce to total stand basal area; Yrs_since last_ = number of years since the last thinning; and $R_{\text{adj}}^{2}$ = adjusted *R*‐squared. Significance levels: “***” 0.001, “**” 0.01, “*” 0.05, “°” 0.1. See Table S7 for the full models.

For severe drought events, APAR and the proportion of spruce were the most important predictors of the analyzed growth responses (Table 3). Stand‐level resistance and resilience were negatively and solely influenced by APAR while recovery was positively affected by higher proportions of spruce (Table 3; Figure 3b).

## 4 DISCUSSION

Based on a unique long‐term silvicultural experiment in mixed silver fir and Norway spruce mountain forests in central Europe, we examined growth responses to mild and severe drought events at both the tree and the stand level. Furthermore, we evaluated the drivers of such responses, using models containing the most influential tree and stand characteristics as well as management‐related variables, to provide insights for the management of such forests under climate change.

### 4.1 Growth response of fir and spruce to drought

**FIGURE 4:**
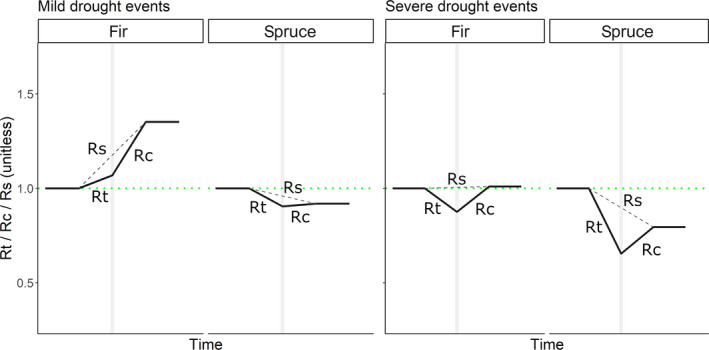
Synthesis scheme of tree‐level growth resistance (Rt), recovery (Rc) and resilience (Rs) of fir and spruce. The graphs are based on the data and overall findings of this study for the two mild (years 1984 and 1991) and severe (years 2003 and 2011) drought events examined. Vertical gray lines denote drought events

Our results highlight the higher drought resistance and resilience of fir compared to spruce, although with variability among sites. This is consistent with other studies showing higher drought tolerance of fir at least in the recent past (e.g., Vitali et al., 2017; Vitasse, Bottero, Cailleret, et al., 2019). It should be noted, however, that in the past centuries this was not necessarily the case: recurring decline events were observed for fir in central Europe in the 19th and 20th centuries, with drought often being considered among the possible major drivers (e.g., Vincent & Kantor, 1971; Wiedemann, 1927). However, the ongoing changes in climate and the recovery of forest soils from a century of depletion (e.g., following litter raking; Ganter, 1927) may have changed relevant ecological conditions that, in turn, positively affected the vigor and resistance of fir. In contrast to fir, spruce exhibited higher recovery to severe drought events. This pattern, however, does not imply a better recovery of spruce compared to fir, but reflects (i) that fir, characterized by higher drought resistance, has less growth to recover after drought compared to spruce (see the synthesis scheme presented in Figure 4) and (ii) the negative nature of the relationship between resistance and recovery that is inherent in the method by Lloret et al. (2011), where the growth during drought is the numerator of the fraction used to calculate resistance, and the denominator for recovery. It has been shown for numerous studies which applied the indicators by Lloret et al. (2011) that forest stands with stronger growth reductions during drought (lower resistance) were indeed capable to recover faster than stands with higher resistance (Schwarz et al., 2020).

In our study, the growth of fir increased during mild drought events compared to previous years without drought stress (resistance >1), and this effect continued after drought (recovery >1). Increased temperatures are the probable cause for the growth increase of fir in relation to these mild drought events. The growth of fir, in fact, likely benefits from warmer conditions, even under low precipitation (Vitasse, Bottero, Rebetez, et al., 2019). Spruce, instead, experienced growth reductions even during mild drought events (resistance <1). Severe drought events indicated clear growth reductions for both species, particularly pronounced for spruce. Spruce is characterized by a smaller hydraulic safety margin and therefore a more risky strategy than fir (Cochard, 1992; Mayr et al., 2003, 2006). If damage occurs in the hydraulic system, water transport is reduced over a longer period even though sufficient water is available (Brodribb et al., 2010). The protracted lower post‐drought growth of spruce may indicate that damage occurred during drought, causing longer‐term legacy effects (cf. Anderegg et al., 2015; Schuldt et al., 2020).

Long‐term growth trends documented for the region of the experiment show that the growth of fir and spruce has been consistently increasing after the growth depression observed in the 1970s. The growth of spruce, however, has restarted to decline from the mid‐1990s (Kohnle et al., 2014; Yue et al., 2011). Our results are in line with the documented trends and the expected future performance of the species: the growth of fir will potentially increase in a warmer climate, in particular if it leads to milder winter and spring conditions (Vitali, Büntgen, et al., 2018), whereas spruce will probably suffer from warmer and drier conditions, with potentially negative repercussions on timber production, especially at lower elevations (Elkin et al., 2013; Pretzsch et al., 2020). Some of the expected difficulties for spruce have become visible with its epochal large‐scale mortality following the extreme summer drought of 2018, which may have profound impacts for the future of European forests and the forestry sector (Hanewinkel et al., 2013; Schuldt et al., 2020).

Finally, we found that stands were overall more resilient than the average individual trees. Stand‐level growth responses to drought are probably buffered by individual growth responses among trees of different species and dimensions, and therefore show averaged responses of lower magnitude than those at the species level. Besides an averaging effect, there is evidence that mixed stands such as those assessed here show complementarity (Ammer, 2019), and the beneficial interactions in admixtures may also convey resilience and adaptability to climate change (Bauhus, Forrester, & Pretzsch, 2017), as observed in mixed stands in temperate forests under drought conditions (Grossiord, Granier, Ratcliffe, et al., 2014).

### 4.2 Predictors of growth responses to mild and severe drought events

#### 4.2.1 Tree level

Species identity was the sole predictor of the tree‐level drought response to mild drought events. In contrast, in the case of severe drought events, additional factors such as drought severity, intraspecific and interspecific interactions and residual stand basal area at the time of drought co‐determined the growth responses to drought.

Less pronounced water deficits (i.e., a higher SPEI value) were associated with higher values of the growth response (particularly recovery and resilience) of fir and spruce to severe drought events (years 2003 and 2011). Drought severity and seasonality are generally important determinants of forest sensitivity (Huang et al., 2018; Li et al., 2020), and can exceed the magnitude of the influence of stand and soil characteristics (D’Orangeville et al., 2018). At our sites, smaller water deficits occurred during the year 2011, which was characterized by dry conditions in late winter and spring. In this case, thus, not only the severity but also the timing of drought may have played a decisive role for the higher recovery and resilience. A global study on the influence of drought timing on post‐drought stem radial growth, in fact, found that extreme droughts during the dry season had larger negative effects on post‐drought tree growth compared to extreme droughts during the wet season (Huang et al., 2018). Besides these effects, we did not find differences in the resilience components among sites that could be caused by differences in mean climatic conditions. For instance, forest resilience has been reported to decrease with increasing mean annual precipitation (Stuart‐Haëntjens et al., 2018), but the climatic gradient covered by our sites is most likely too narrow to confirm this trend.

Interspecific interactions (mainly with fir and beech) negatively affected the drought resistance of spruce during severe droughts. Broadleaf species such as beech are characterized during the growing season by a high interception of precipitation, stemflow (channel water more to their own rooting system), and may therefore play a negative role especially during periods of water shortage (Staelens et al., 2008). Beyond these aboveground interactions, it is unclear to what extent the drought response of spruce may be driven by changes in rooting depth and intensity in mixtures when compared to pure stands. Observations from beech and spruce mixtures in Austria and Germany showed that the root system of spruce tends to be confined to the upper soil layers, suffering from strong competition by beech (Bolte & Villanueva, 2006; Schume et al., 2004). Another study conducted in southern Germany supported these findings, but found no increase in drought stress or growth reduction of spruce mixtures with beech (Goisser et al., 2016). The pattern observed in our study suggests that spruce subjected to strong interspecific interaction may suffer from higher competition for resources, which would further lower soil moisture availability during droughts, as observed in boreal forests (Grossiord, Granier, Gessler, et al., 2014). In contrast, our results suggest that facilitation and/or resource partitioning may underlie the improved growth resistance of fir subjected to interspecific interaction (cf. Vitali, Forrester, et al., 2018).

The recovery of fir following severe drought events was negatively affected by the presence of other species, including a higher presence of spruce. After a drought, it takes time until deeper soil layers are rewetted. Species with a shallow root system, such as spruce, are thus likely to profit first, at the expense of coexisting species with deeper root systems (Schume et al., 2004). Complementarity in the temporal origin of water used by spruce and beech, which has been observed in temperate forests (Brinkmann et al., 2018), could explain the positive effect of other species (mainly beech) on the recovery to drought of spruce found in our study.

The contrasting effects of species diversity on tree growth resilience to severe droughts observed here and elsewhere show that these effects in mixed stands are often complex and depend strongly on the local environment and the complementarity of species‐specific functional traits (Ammer, 2019; Ratcliffe et al., 2017).

Stand‐level competition directly affects resource availability (Moreno‐Gutiérrez et al., 2012). High stand density (i.e., basal area) is often associated with higher rainfall interception and transpiration (van Dijk & Bruijnzeel, 2001), thus lowering soil water availability and storage (Bréda et al., 1995), as observed in several Norway spruce stands in Europe (e.g., Misson et al., 2003; Sohn et al., 2013) and various species worldwide (e.g., Andrews et al., 2020; Navarro‐Cerrillo et al., 2019; Sohn, Hartig, et al., 2016). However, other studies reported a minor or variable influence of residual stand basal area on the growth response to drought (e.g., Serra‐Maluquer et al., 2018; Sohn et al., 2016). We found a negative influence of residual stand basal area on the tree‐level response to severe droughts, which was significant for the resistance of fir and the resilience of both species, but rather marginal (effect size <7%). The magnitude of the effect of residual basal area on the drought response is not usually examined in studies of this kind. However, the relatively limited effect of residual stand basal area that we observed may be related to local conditions and stand characteristics. At the experimental stands, the relatively narrow range of stand basal area that was removed with each intervention may mask the potential beneficial effect of stand density reduction, as it was clearly observed under more pronounced density reductions (e.g., Sohn et al., 2013).

#### 4.2.2 Stand level

Stand structural characteristics and composition, and years since the last thinning determined growth responses to drought at the stand level.

Stands with larger and taller trees with larger leaf areas (i.e., higher APAR, Figure S11) had higher recovery following mild drought events. Trees growing in mesic and fertile locations, like our study sites, can develop large crowns. Often it is assumed that tall stature and large crowns make trees more susceptible to drought (Bennett et al., 2015; Grote et al., 2016). During the mild droughts assessed here, the positive effects of a large crown (i.e., the provisioning of photosynthates) outweighed potential negative effects (e.g., higher water loss). Furthermore, abundant carbohydrate reserves of these trees may support post‐drought growth (Zweifel et al., 2020), therefore improving the overall resilience of stands on productive sites. For example, individual Scots pine trees growing at low‐productivity sites across Europe showed lower drought recovery and resilience (Bose et al., 2020). In contrast to the situation under mild droughts, in our study the same stands were less resistant and resilient to severe droughts. The higher drought vulnerability of larger and taller trees is caused by higher radiation and evaporative demand of the exposed crowns, and a greater inherent vulnerability to xylem embolism (Bennett et al., 2015; Olson et al., 2018). Thus, the lower resilience may be related to the post‐drought necessity of large trees to restore their hydraulic system and crown (Choat et al., 2018), thus slowing recovery (Brodribb et al., 2010).

We found that resistance to mild droughts was higher in the immediate post‐intervention period. A possible explanation is that the first interventions at the beginning of the experiment led to a more intense competition release than later interventions, when the stands had already been thinned multiple times (Giuggiola et al., 2016; Simon et al., 2017). Additionally, younger trees can respond more strongly with crown expansion to release than older trees (Nyland, 2016).

### 4.3 Implications for forest management in the face of climate change

The cumulative effects of severe droughts and species composition are likely to have major consequences for mixed fir and spruce forests in central Europe. This study highlights the importance of stand composition for tree‐ and stand‐level drought responses. With adequate water supply and in the absence of disturbances, spruce is more productive than fir and most other species under similar site conditions (Pretzsch, 2005). However, as the growth of spruce under current environmental and climatic conditions is generally less resilient to drought than fir, and biotic and abiotic disturbances are likely to increase with climate change, the productivity of this species may be strongly reduced in the future (Seidl et al., 2017; Temperli et al., 2013). The unprecedented tree mortality triggered by the 2018/2019 drought and the associated bark beetle outbreaks in central European temperate forests (Schuldt et al., 2020) emphasize the importance of developing appropriate adaptation strategies. Reducing the proportion of spruce, for instance by fostering mixtures with more drought‐tolerant broadleaf species, and/or by reducing stand density, would improve the resilience of these forests to future (extreme) droughts and other disturbances, with positive repercussions on the provision of multiple ecosystem services (Bauhus, Forrester, & Pretzsch, 2017). In addition, management strategies aiming for smaller size trees would be beneficial for the resilience of trees and stands to drought, as taller trees are more vulnerable to hydraulic stress (Grote et al., 2016) and larger spruce trees are also more susceptible to bark beetle infestations (Netherer & Nopp‐Mayr, 2005). Our study demonstrates that interventions at short intervals can improve the drought resilience of mature stands, especially in dense stands and during mild droughts.

Although the growth response to drought has been investigated for many tree species at the individual tree level, much remains unknown about the drought response of forest stands. This lack of knowledge results because many studies do not sample the full range of tree sizes, and are not based on random samples of trees. This inhibits the scaling of growth from individual trees across species and dimensions to whole stands. The scaling tasks is further complicated by the fact that typically there are no data for trees that have died or were removed over time, which is particularly relevant in managed forests or when the analysis extends over long periods.

Overall, the mixed silver fir and Norway spruce stands in central Europe that we studied are resilient to mild drought events, and even profit from such conditions, but they suffer from severe droughts. Forest management can support these forests by promoting their growth resistance, recovery and resilience, specifically by controlling or modifying species composition, tree size distribution and stand density. The approach described here provides relevant information for the management of the widespread European mixed mountain forests dominated by fir and spruce in the face of climate change.

## CONFLICT OF INTEREST

The authors declare no conflict of interest.

## Supporting information

Supplementary Material

## Data Availability

The data that support the findings of this study are available from the corresponding author upon reasonable request.
