# Supplementary material for: Growth resistance and resilience of mixed silver fir and Norway spruce forests in central Europe: Contrasting responses to mild and severe droughts
Source: Glob Chang Biol. 2021 Jun 24;27(18):4403–19. doi: 10.1111/gcb.15737 (PMC8453522; doi:10.1111/gcb.15737)
Supplement: Supplementary file 1 — Supplementary Material [file GCB-27--s001.pdf]

Bottero et al. **"Growth resistance and resilience of mixed silver fir and Norway spruce forests in central Europe – contrasting responses to mild and severe droughts"**

SUPPORTING INFORMATION

Appendix S1: Extended materials and methods information.

Appendix S2: Supplementary references.

Table S1: Tree- and stand-level variables tested to model resistance, resilience and recovery to drought.

Table S2: Parameters used for the Maestra model.

Table S3: Optimal random structure selection based on AIC and likelihood ratio test.

Table S4: Differences in drought responses among drought events, forest components and treatments (Kruskal-Wallis tests).

Table S5: Differences in drought responses among drought events, forest components and treatments (pairwise comparisons).

Table S6: Linear mixed-effect models. Fit of tree-level resistance, recovery, and resilience as function of different variables (full models).

Table S7: Linear mixed-effect models. Fit of stand-level resistance, recovery, and resilience as function of different variables (full models).

Figure S1: Tree stem density across treatments and sites since 1980.

Figure S2: Correlation between SPEI and tree-ring chronologies using different time scales for the SPEI.

Figure S3: SPEI of July at the time scale of 5 months for the period 1980-2016.

Figure S4: Smoothed density estimates of tree-level drought resistance, recovery and resilience across sites, treatments and species, for the period 1980-2016.

Figure S5: Smoothed density estimates of stand-level drought resistance, recovery and resilience across sites and treatments, for the period 1980-2016.

Figure S6: Radial growth autocorrelation.

Figure S7: Scatter plot of tree-level variables with correlation coefficients.

Figure S8: Scatter plot of stand-level variables with correlation coefficients.

Figure S9: Mean-value site chronology (tree-ring width indices) of fir and spruce across treatments and sites since 1980.

Figure S10: Boxplots of resistance, recovery and resilience by forest component, drought event and treatment.

Figure S11: Relationships between APAR and tree characteristics.

Appendix S1: Extended materials and methods information

Study sites

The shelterwood experiment (Weise, 1995) comprised three treatments differing in length of the regeneration period (20, fast, 35, medium, and 50 years, slow) and increment controls (stands maintained fully stocked by harvesting only 50% of the periodic increment every 5 years). The stands used in the experiment did not receive interventions in the 10 years preceding the beginning of the experiment (initiated between 1979 and 1981); the treatments were assigned to approximately 0.25 ha square plots. The three treatments were cut to 75% of the volume of a fully stocked stand at the time of the research installation. The interventions were planned at 5-year intervals in each treatment, according to the following scheme:

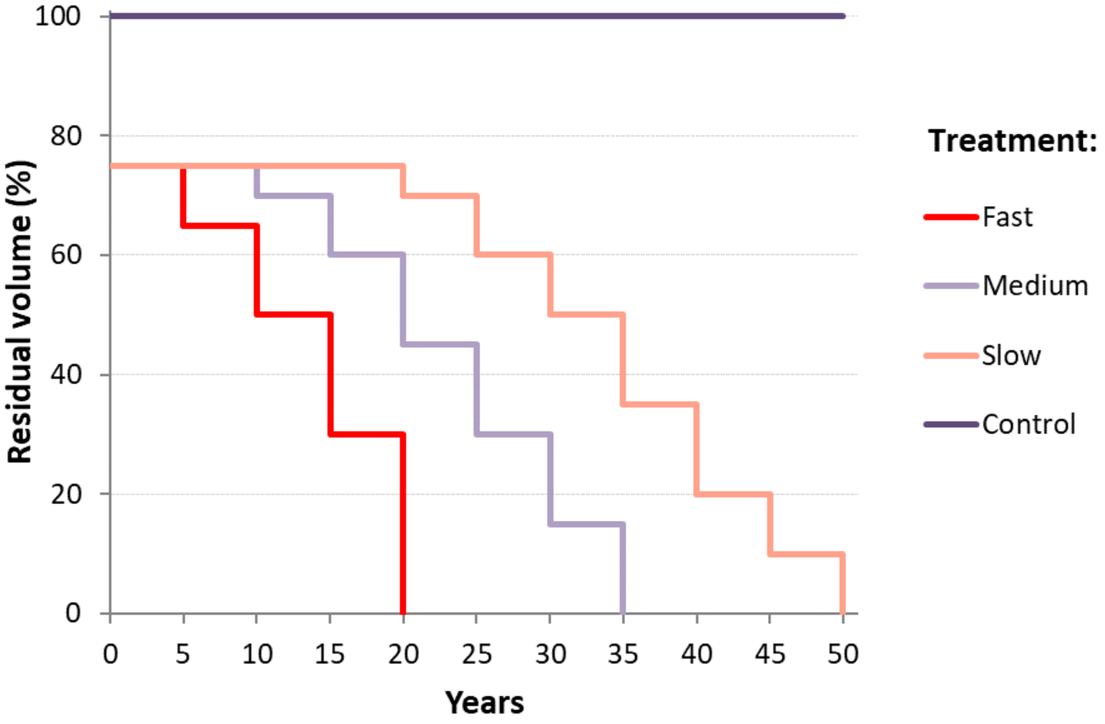

Note that the fast treatment (20-year regeneration period, ended in the early 2000s) did not cover the entire period of analysis, and thus was not included in the analysis presented in this study.

Inventory and field data collection, and laboratory analysis

The diameter at breast height (DBH), height, live crown length, crown radii and leaf area of the trees for which these variables were not measured, were predicted from the sampled trees using the following equations (Forrester et al., 2019), for each plot and species:

$$DBH_t = \beta_0 + \beta_1 DBH_{t-1} \quad (1)$$

where DBH_t is DBH at year t in cm, DBH_{t-1} is DBH of the previous year (t-1) in cm and β_0 and β_1 are fitted parameters.

$$y = 1.3 + \beta_0 e^{-\beta 1 / DBH} \quad \text{(Michajlov, 1952)} \quad (2)$$

where y is total height in m or live crown length in m, DBH in cm and β_0 and β_1 are fitted parameters.

$$ln(KRA) = \beta_0 + \beta_1 ln(DBH) \quad (3)$$

where KRA is crown radius in m, DBH in cm and β_0 and β_1 are fitted parameters.

Leaf area values were obtained using species-specific leaf area allometric equations (Forrester et al., 2017), where leaf area is predicted from DBH.

Statistical analyses

Twelve full models (2 levels: tree, stands; 2 drought groups: mild, severe; and 3 responses: resistance, recovery, resilience) were used to test different random structures to select the optimal random structure and, thus, type of model for analysis: random intercept to account for variability in the growth response to drought among trees within the same plot (tree-level models), and among plots within the same site (stand-level models); random intercept and slope, containing residual stand basal area as a fixed effect with a random slope and intercept; and no random term (Table S3). The restricted maximum likelihood method was used to evaluate the optimal random structure of the full models (Zuur et al., 2009).

Appendix S2: Supplementary references

Allen, R. G., Pereira, L. S., Raes, D., & Smith, M. (1998). Crop evapotranspiration-Guidelines for computing crop water requirements-FAO Irrigation and drainage paper 56. *Fao, Rome, 300*(9), D05109.

Bunn, A., Korpela, M., Biondi, F., Campelo, F., Mérian, P., Mudelsee, M., Qeadan, F., Schulz, M., & Zang, C. (2014). dplR: Dendrochronology Program Library in R. *R Package Version 1.6.0*. http://CRAN.R-project.org/package=dplR

Dietrich, L., Zweifel, R., & Kahmen, A. (2018). Daily stem diameter variations can predict the canopy water status of mature temperate trees. *Tree Physiology*.

Forrester, D. I. (2019). Linking forest growth with stand structure: Tree size inequality, tree growth or resource partitioning and the asymmetry of competition. *Forest Ecology and Management*, *447*, 139–157.

Forrester, D. I., Nitzsche, J., & Schmid, H. (2019). *The Experimental Forest Management project: An overview and methodology of the long-term growth and yield plot network*. Swiss Federal Institute of Forest, Snow and Landscape Research WSL.

Forrester, D. I., Tachauer, I. H. H., Annighoefer, P., Barbeito, I., Pretzsch, H., Ruiz-Peinado, R., Stark, H., Vacchiano, G., Zlatanov, T., & Chakraborty, T. (2017). Generalized biomass and leaf area allometric equations for European tree species incorporating stand structure, tree age and climate. *Forest Ecology and Management*, *396*, 160–175.

Kolebaje, O. T., & Mustapha, L. O. (2012). On the performance of some predictive models for global solar radiation estimate in tropical stations: Port Harcourt and Lokoja. *The African Review of Physics*, *7*(15), 145–163.

Michajlov, J. (1952). Matematische Formulierung des Gesetzes für Wachstum und Zuwachs der Waldbäume und Bestände. *Schweiz. Z. Forstw*, *103*(9), 10.

Weise, U. (1995). *Zuwachs-und Jungwuchsentwicklung in Versuchen zur natürlichen Verjüngung von Fichten-Tannen (Buchen)-Beständen in Baden-Württemberg* (No. 192; Mitteilungen der Forstlichen Versuchs- und Forschungsanstalt Baden-Württemberg, p. 75). Forstliche Versuchs-und Forschungsanst. Baden-Württemberg.

Zang, C., & Biondi, F. (2013). Dendroclimatic calibration in R: the bootRes package for response and correlation function analysis. *Dendrochronologia*, *31*(1), 68–74.

Zuur, A., Ieno, E. N., Walker, N., Saveliev, A. A., & Smith, G. M. (2009). *Mixed effects models and extensions in ecology with R*. Springer Science & Business Media.

Table S1: Summary of tree- and stand-level variables tested to model resistance, recovery and resilience to drought. APAR = absorption of photosynthetically active radiation; NI = competition index.

			1984	1991	2003	2011
**Level**	**Name**	**Description**	**Mean (sd) Range**	**Mean (sd) Range**	**Mean (sd) Range**	**Mean (sd) Range**
Tree	DIB	Diameter at breast height inside bark (cm)	33.54(12.61) 4.13-66.17	37.55 (13.22) 5.50-71.06	41.07(14.54) 7.86-82.13	43.86(14.92) 8.57-78.90
Tree	H	Total tree height (m)	25.94(7.47) 2.40-37.93	27.40(7.62) 2.57-39.34	27.97(8.45) 3.51-39.93	28.80(7.79) 4.88-40.41
Tree	APAR	Total tree APAR (GJ/tree/year)	62.07(42.62) 0.55-227.88	73.04(47.29) 1.81-247.05	88.32(55.13) 3.22-249.16	100.78(63.76) 6.16-286.00
Tree	NI_tot	NI all species (cm^2/m)	1276.87(564.20) 0.00-3883.00	1281.06(612.27) 0.00-4236.80	1152.80(714.49) 0.00-4037.70	1095.52(854.76) 0.00-4137.10
Tree	NI_fir	NI fir (%)	0.48(0.32) 0.00-1.00	0.49(0.33) 0.00-1.00	0.51(0.35) 0.00-1.00	0.56(0.36) 0.00-1.00
Tree	NI_spruce	NI spruce (%)	0.43(0.33) 0.00-1.00	0.43(0.34) 0.00-1.00	0.38(0.35) 0.00-1.00	0.33(0.35) 0.00-1.00
Tree	NI_other	NI other species (%)	0.09(0.15) 0.00-1.00	0.08(0.15) 0.00-1.00	0.12(0.20) 0.00-1.00	0.11(0.20) 0.00-1.00
Tree, Stand	BA_stand	Total stand basal area remaining (m^2/ha)	29.24(4.84) 21.73-40.33	30.19(6.31) 24.08-46.70	27.81(10.75) 11.46-52.10	27.73(15.18) 7.02-56.33
Tree, Stand	SPEI	SPEI July 5 months (unitless)	-0.59(0.61) -1.27-0.42	-0.45(0.30) -1.01- -0.11	-2.24(0.26) -2.51- -1.72	-1.68(0.25) -1.95- -1.25
Stand	m_H	Mean stand height (m)	26.75(3.85) 19.01-31.59	27.86(3.96) 20.74-32.83	28.93(5.16) 19.76-34.88	29.25(5.35) 19.66-35.34
Stand	m_DBH	Mean stand DBH (cm)	34.87(5.43) 24.85-44.10	38.50(5.48) 28.97-48.46	43.09(7.68) 30.10-54.52	45.66(9.71) 29.45-62.19
Stand	Shannon	Shannon diversity index	0.72(0.17) 0.39-1.04	0.72(0.16) 0.40-1.02	0.76(0.14) 0.55-1.06	0.71(0.25) 0.00-1.07
Stand	Fir	Ratio of basal area of fir to stand basal area (%)	0.51(0.21) 0.18-0.89	0.51(0.21) 0.18-0.89	0.54(0.17) 0.24-0.81	0.60(0.18) 0.34-1.00
Stand	Spruce	Ratio of basal area of spruce to stand basal area (%)	0.43(0.22) 0.08-0.82	0.43(0.23) 0.09-0.82	0.38(0.21) 0.12-0.76	0.35(0.17) 0.11-0.61
Stand	other	Ratio of basal area of other species to stand basal area (%)	0.08(0.09) 0.00-0.21	0.09(0.08) 0.00-0.21	0.13(0.09) 0.01-0.28	0.13(0.10) 0.02-0.29
Stand	BA_removed	Stand basal area removed (m^2/ha)	1.30(1.31) 0.17-3.99	2.82(1.52) 0.23-4.87	3.50(3.40) 0.33-10.57	5.90(2.39) 1.11-9.07
Stand	BA_removed_cum	Cumulative sum of stand basal area removed (m^2/ha)	2.74(2.36) 0.17-7.28	6.09(1.62) 3.55-9.46	17.88(5.30) 9.82-26.38	27.27(8.34) 13.55-39.53
Stand	N_thin	Total number of interventions	1.73(0.90) 1-3	3.56(1.41) 2-7	6.56(1.59) 4-9	8.56(1.41) 7-11
Stand	yrs_since_last	Number of years since the last thinning	2.18(1.40) 1-5	2.88(2.28) 1-9	3.25(1.34) 1-5	2.31(1.40) 1-5

Table S2: Summary of site- and species-specific parameters used for the Maestra model.

Site-specific

	Ta 220	Ta 221	Ta 222	Ta 223	Ta 224	Ta 225
Mean leaf unfolding[1] (Julian day) 1980-2015	135	109	113	113	117	115
Mean autumnal coloring of leaves (50%)[1] (Julian day) 1980-2015	278	284	257	257	279	274

Species-specific (Forrester, 2019)

	Leaf transmittance in PAR/NIR/thermal	Leaf reflectance in PAR/NIR/thermal	Parameters (a / b / c) for the vertical leaf area density (beta distribution: BPT)	Mean leaf inclination angle
Abies alba	0.03 / 0.26 / 0.00	0.09 / 0.33 / 0.05	13.68 / 1.22 / 1.84	10
Fagus sylvatica	0.05 / 0.30 / 0.05	0.06 / 0.35 / 0.05	0.57 / 0.04 / -0.45	21
Picea abies	0.03 / 0.26 / 0.00	0.09 / 0.33 / 0.05	13.68 / 1.22 / 1.84	30

References: [1]PEP725 Pan European Phenology Data. Data set accessed 2018-04-20 at http://www.pep725.eu/

Photosynthetically active radiation (PAR) was computed using two different Angstrom equations to get the solar radiation, then averaged. One equation uses sunshine hours (Allen et al., 1998) and the other uses the difference between maximum and minimum temperature (Kolebaje & Mustapha, 2012).

Table S3: Summary of optimal random structure selection based on AIC and likelihood ratio test. Random structures tested: no random term (noR), random intercept model using site/plot (tree-level) or site (stand-level) (Ri), and random intercept and slope model (Ris). Rt = resistance, Rc = recovery, Rs = resilience, mild = mild drought events (1984 and 1991), severe = severe drought events (2003 and 2011). The *p*-values were corrected to deal with testing on the boundary (Zuur et al., 2009).

Tree-level

	Model	df	AIC	BIC	logLik	Test	L.Ratio	*p*-value	Correct *p*-value
**mild**									
Rt	noR	13	1414.14	1468.59	-694.07				
Rt	Ri	15	1396.36	1459.19	-683.18	1 vs 2	21.78	0.000	0.000
Rt	Ris	19	1398.74	1478.32	-680.37	2 vs 3	5.62	0.230	0.039
Rc	noR	13	1436.64	1491.08	-705.32				
Rc	Ri	15	1438.11	1500.94	-704.06	1 vs 2	2.52	0.283	0.056
Rc	Ris	19	1444.64	1524.22	-703.32	2 vs 3	1.47	0.831	0.352
Rs	noR	13	1396.99	1451.43	-685.49				
Rs	Ri	15	1391.06	1453.88	-680.53	1 vs 2	9.93	0.007	0.001
Rs	Ris	19	1395.36	1474.94	-678.68	2 vs 3	3.70	0.448	0.106
**severe**									
Rt	noR	13	723.43	769.32	-348.72				
Rt	Ri	15	705.09	758.04	-337.55	1 vs 2	22.34	0.000	0.000
Rt	Ris	19	713.09	780.15	-337.55	2 vs 3	0.00	1.000	0.994
Rc	noR	13	648.00	691.32	-311.00				
Rc	Ri	15	644.27	694.26	-307.14	1 vs 2	7.73	0.021	0.003
Rc	Ris	19	652.27	715.60	-307.14	2 vs 3	0.00	1.000	0.995
Rs	noR	13	611.89	655.22	-292.95				
Rs	Ri	15	614.34	664.34	-292.17	1 vs 2	1.55	0.461	0.107
Rs	Ris	19	622.34	685.67	-292.17	2 vs 3	0.00	1.000	0.995
**Stand-level**									
**mild**									
Rt	noR	9	105.57	116.89	-43.79				
Rt	Ri	10	107.15	119.73	-43.57	1 vs 2	0.43	0.514	0.257
Rt	Ris	12	111.04	126.14	-43.52	2 vs 3	0.10	0.949	0.848
Rc	noR	9	114.99	126.31	-48.49				
Rc	Ri	10	115.95	128.53	-47.97	1 vs 2	1.04	0.308	0.154
Rc	Ris	12	119.95	135.05	-47.98	2 vs 3	0.00	0.999	0.981
Rs	noR	9	113.21	124.53	-47.60				
Rs	Ri	10	114.73	127.31	-47.36	1 vs 2	0.48	0.488	0.244
Rs	Ris	12	118.62	133.72	-47.31	2 vs 3	0.10	0.950	0.850
**severe**									
Rt	noR	9	110.54	120.76	-46.27				
Rt	Ri	10	112.29	123.65	-46.15	1 vs 2	0.24	0.621	0.310
Rt	Ris	12	114.98	128.60	-45.49	2 vs 3	1.31	0.518	0.385
Rc	noR	9	93.32	101.82	-37.66				
Rc	Ri	10	95.30	104.74	-37.65	1 vs 2	0.03	0.864	0.432
Rc	Ris	12	99.04	110.37	-37.52	2 vs 3	0.25	0.881	0.747
Rs	noR	9	91.69	100.19	-36.84				
Rs	Ri	10	93.61	103.05	-36.80	1 vs 2	0.08	0.784	0.392
Rs	Ris	12	94.97	106.30	-35.48	2 vs 3	2.64	0.267	0.186

Table S4: Summary of differences in drought responses among drought events, forest components and treatments. Results are χ^2 following a Kruskal-Wallis rank sum test. Significance levels: '****' 0.0001, '***' 0.001, '**' 0.01, '*' 0.05, 'ns' not significant.

	**Levels**	**Resistance**	**Recovery**	**Resilience**
**Drought event**	Mild, Severe	146.94****	5.48*	69.19****
**Forest component**	Spruce, Fir, Stand (mild)	48.69****	41.18****	106.90****
	Spruce, Fir, Stand (severe)	63.87****	14.72***	47.45****
**Treatment**	Control, Slow, Medium (mild)	5.72 ns	3.11 ns	9.24**
	Control, Slow, Medium (severe)	6.85*	1.95 ns	0.88 ns

Table S5: Summary of differences in drought responses among drought events, forest components and treatments. Results are adjusted *p*-values following a pairwise comparison using the non-parametric Wilcoxon rank sum test and a Bonferroni correction for multiple testing.

		**Resistance**	**Recovery**	**Resilience**
**Drought event**	Mild – Severe	< 0.0001	0.0192	< 0.0001
**Forest component**	Fir – Spruce (mild)	0.0960	0.0780	0.0002
	Fir – Stand (mild)	0.2160	1.0000	0.0600
	Spruce – Stand (mild)	< 0.0001	< 0.0001	< 0.0001
	Fir – Spruce (severe)	0.8700	0.0300	1.0000
	Fir – Stand (severe)	0.0003	1.0000	< 0.0001
	Spruce – Stand (severe)	< 0.0001	0.0030	< 0.0001
**Treatment**	Control – Slow (mild)	1.0000	1.0000	0.7920
	Control – Medium (mild)	1.0000	1.0000	1.0000
	Slow – Medium (mild)	0.1080	0.6000	0.0180
	Control – Slow (severe)	0.2220	1.0000	1.0000
	Control – Medium (severe)	0.0720	1.0000	1.0000
	Slow – Medium (severe)	1.0000	1.0000	1.0000

Table S6: Summary of linear mixed-effect models. Fit of tree-level resistance, recovery, and resilience for mild (years 1984 and 1991) and severe drought events (years 2003 and 2011) as a function of different variables (full models). Sp = species (2 levels: fir, reference spruce); APAR = absorption of photosynthetically active radiation; $NI_{ratio\ fir}$ = ratio of intensity of competition of fir to total intensity of competition; $NI_{ratio\ other}$ = ratio of intensity of competition of other species (mainly beech) to total intensity of competition; BA_{stand} = stand basal area; SPEI = SPEI of July at the time scale of 5 months; x = interaction; R^2_m = marginal R-squared (variance explained by the fixed factors); and R^2_c = conditional R-squared (variance explained by the fixed and random factors). Significance codes: '***' 0.001, '**' 0.01, '*' 0.05, '°' 0.1.

**Tree-level**	**Full model**	**Full model**	**Full model**
	**Estimate (se)**	**Estimate (se)**	**Estimate (se)**
**Mild**	**Resistance**	**Recovery**	**Resilience**
Intercept	-0.45(0.16)*	-0.40(0.12)**	-0.53(0.14)**
Sp_{fir}	0.72(0.11)***	0.60(0.11)***	0.84(0.10)***
APAR	-0.03(0.07)	0.04(0.07)	0.01(0.07)
$NI_{ratio\ fir}$	0.06(0.09)	-0.04(0.09)	0.02(0.09)
NI_{ratio_other}	-0.14(0.10)	0.14(0.10)	0.03(0.10)
BA_{stand}	0.01(0.08)	-0.10(0.09)	-0.07(0.08)
SPEI	-0.07(0.06)	0.07(0.06)	0.04(0.06)
APAR x Sp_{fir}	-0.05(0.10)	-0.01(0.11)	-0.04(0.10)
$NI_{ratio\ fir}$ x Sp_{fir}	-0.11(0.11)	0.18(0.11)	0.05(0.10)
NI_{ratio_other} x Sp_{fir}	0.11(0.11)	-0.13(0.11)	-0.06(0.11)
BA_{stand} x Sp_{fir}	-0.08(0.10)	0.09(0.11)	0.02(0.10)
R^2_m	0.142	0.109	0.177
R^2_c	0.235	0.142	0.244
**Severe**	**Resistance**	**Recovery**	**Resilience**
Intercept	-0.70(0.19)**	0.13(0.19)	-0.57(0.12)***
Sp_{fir}	1.15(0.13)***	-0.36(0.15)*	0.86(0.14)***
APAR	-0.02(0.11)	-0.02(0.14)	0.03(0.12)
$NI_{ratio\ fir}$	-0.21(0.10)*	-0.01(0.12)	-0.28(0.11)**
NI_{ratio_other}	-0.30(0.11)**	0.46(0.13)***	-0.02(0.11)
BA_{stand}	0.03(0.14)	-0.05(0.14)	0.00(0.13)
SPEI	0.15(0.07)*	0.26(0.08)**	0.27(0.07)***
APAR x Sp_{fir}	-0.10(0.15)	0.00(0.18)	-0.17(0.17)
$NI_{ratio\ fir}$ x Sp_{fir}	0.32(0.13)*	-0.12(0.15)	0.25(0.14)°
NI_{ratio_other} x Sp_{fir}	0.34(0.13)**	-0.51(0.15)***	0.04(0.14)
BA_{stand} x Sp_{fir}	-0.35(0.14)*	0.05(0.17)	-0.30(0.15)°
R^2_m	0.346	0.186	0.262
R^2_c	0.507	0.295	0.294

Table S7: Summary of linear regression models. Fit of stand-level resistance, recovery, and resilience for mild (years 1984 and 1991) and severe drought events (years 2003 and 2011) as a function of different variables (full models). APAR = absorption of photosynthetically active radiation; Shannon = Shannon diversity index; $Ratio_{spruce}$ = ratio of basal area of spruce to total stand basal area; BA_{stand} = stand basal area; DBH = mean diameter at breast height (1.3 m height); $Yrs_{since\ last}$ = number of years since the last thinning; SPEI = SPEI of July at the time scale of 5 months; and R^2_{adj} = adjusted R-squared. Significance codes: '***' 0.001, '**' 0.01, '*' 0.05, '°' 0.1.

**Stand-level**	**Full model**	**Full model**	**Full model**
	**Estimate (se)**	**Estimate (se)**	**Estimate (se)**
**Mild**	**Resistance**	**Recovery**	**Resilience**
Intercept	0.00(0.15)	0.00(0.19)	0.00(0.18)
APAR	-0.06(0.24)	0.42(0.30)	0.30(0.28)
Shannon	-0.76(0.23)**	0.05(0.27)	-0.33(0.26)
$Ratio_{spruce}$	0.11(0.19)	-0.54(0.23)*	-0.35(0.22)
BA_{stand}	0.13(0.19)	0.12(0.24)	0.14(0.23)
$Yrs_{since\ last}$	-0.30(0.19)	-0.16(0.23)	-0.27(0.22)
SPEI	-0.13(0.19)	-0.09(0.23)	-0.13(0.22)
R^2_{adj}	0.375*	0.067	0.140
**Severe**	**Resistance**	**Recovery**	**Resilience**
Intercept	0.00(0.22)	0.00(0.20)	0.00(0.22)
APAR	-0.41(0.35)	0.16(0.32)	-0.30(0.35)
Shannon	-0.04(0.34)	-0.03(0.31)	0.01(0.34)
$Ratio_{spruce}$	-0.07(0.24)	0.33(0.23)	0.06(0.25)
BA_{stand}	-0.01(0.29)	0.30(0.26)	0.13(0.29)
$Yrs_{since\ last}$	0.15(0.29)	-0.39(0.27)	0.05(0.30)
SPEI	-0.04(0.28)	-0.28(0.26)	-0.11(0.28)
R^2_{adj}	0.104	0.059	0.141

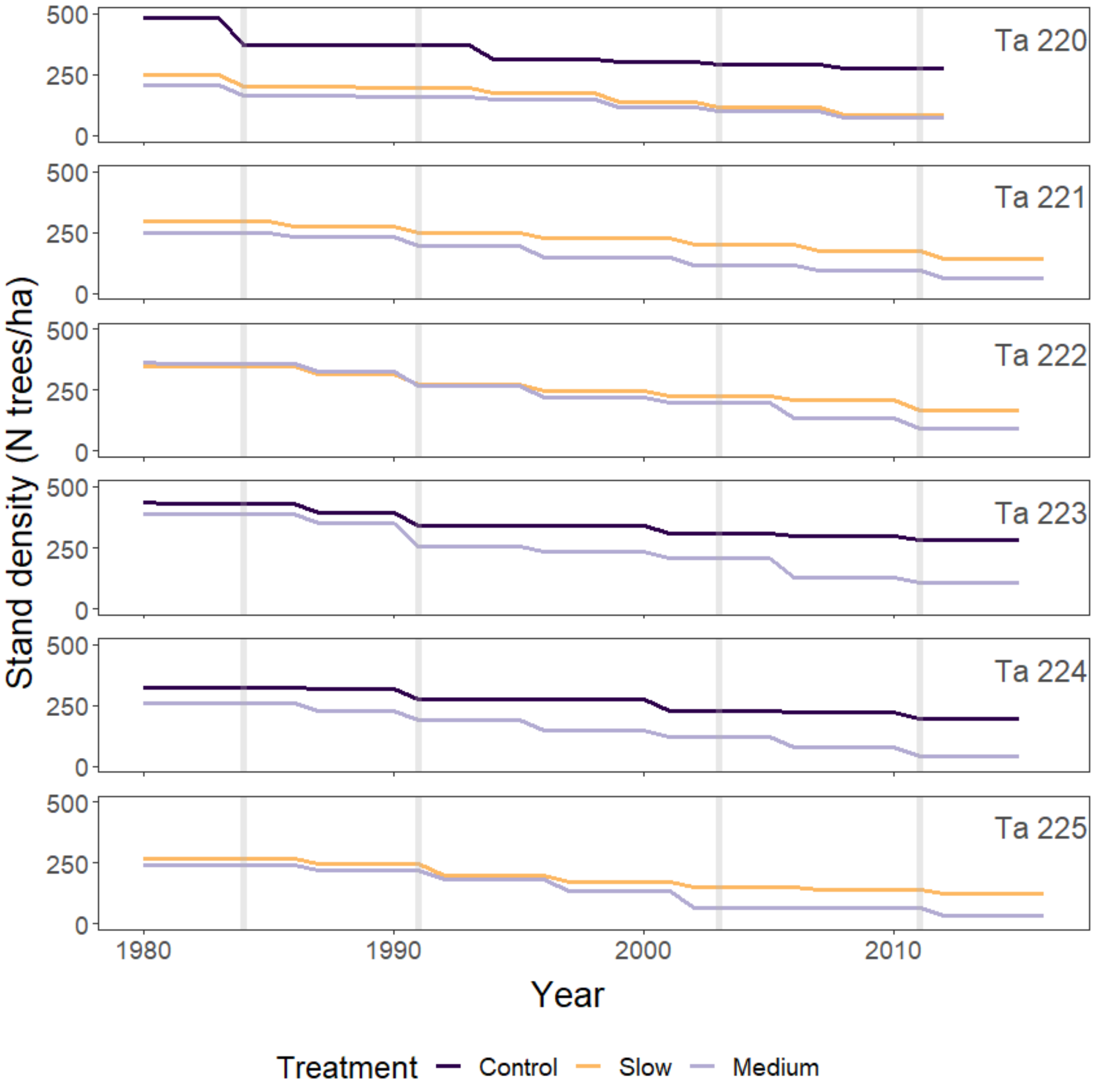

Figure S1: Tree stem density (N trees/ha) across treatments and sites since 1980. Vertical grey lines denote the years 1984, 1991, 2003, and 2011.

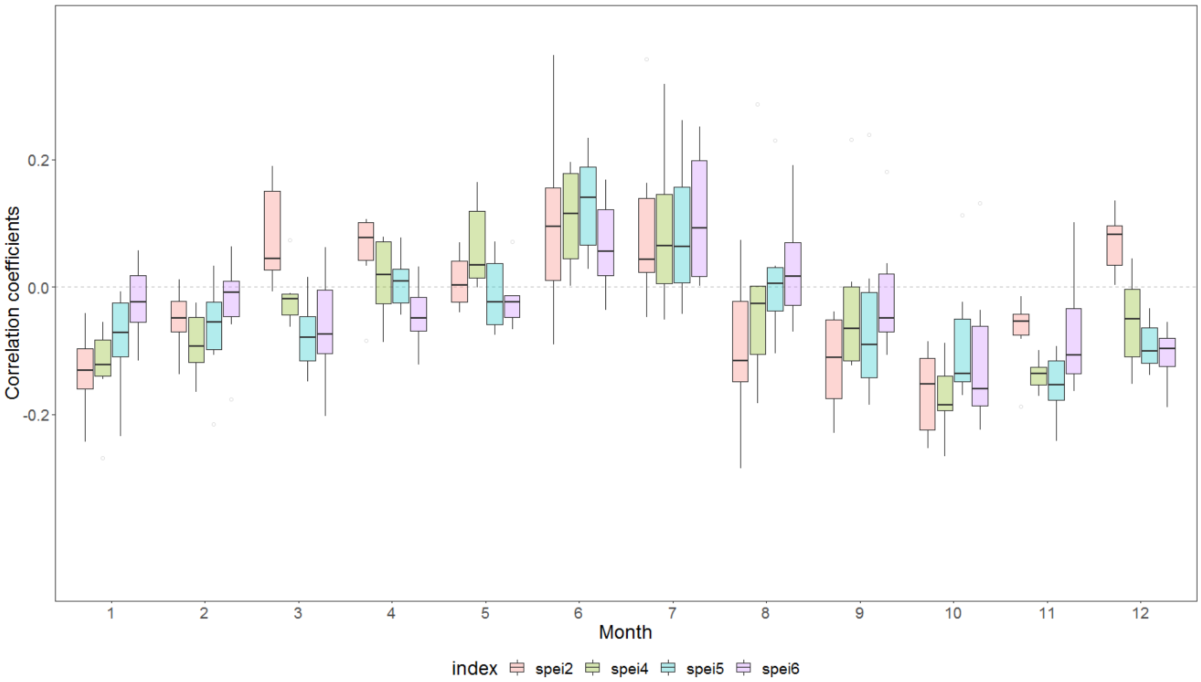

Figure S2: Correlation between SPEI and tree-ring chronologies using different time scales for the SPEI (2, 4, 5, and 6 months). No significant differences were found among the four SPEI in and between the months of June and July (repeated ANOVA tests, $\alpha > 0.05$). Therefore, SPEI of July at the time scale of 5 months was selected because it covers the period of most radius increment for trees in the area (Dietrich et al., 2018). Correlation coefficients were calculated using the function *dcc* of the R package *bootRes* (Zang & Biondi, 2013). Individual tree-ring series were detrended by a smoothing spline, with 50% frequency response at 2/3 of series' length. Site chronologies were built using the Tukey's biweight robust mean with the function *tbrm* of the R package *dplR* (Bunn et al., 2014).

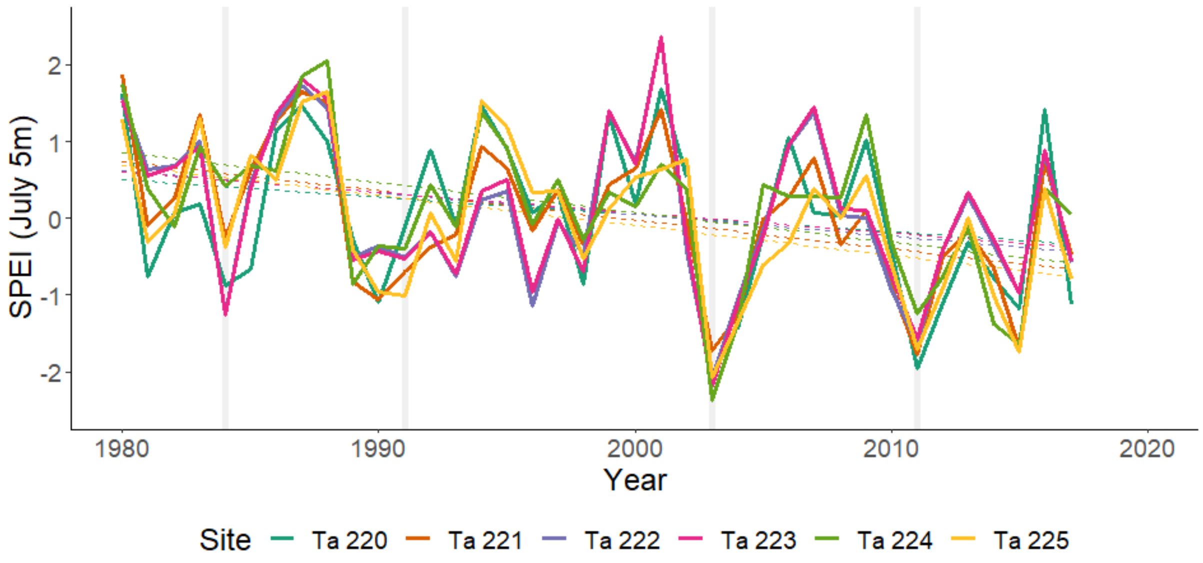

Figure S3: SPEI of July at the time scale of 5 months (SPEI July 5m) for the period 1980-2016 across the study sites; dotted lines show temporal trends of the index at each site; vertical grey lines denote the years 1984, 1991, 2003, and 2011.

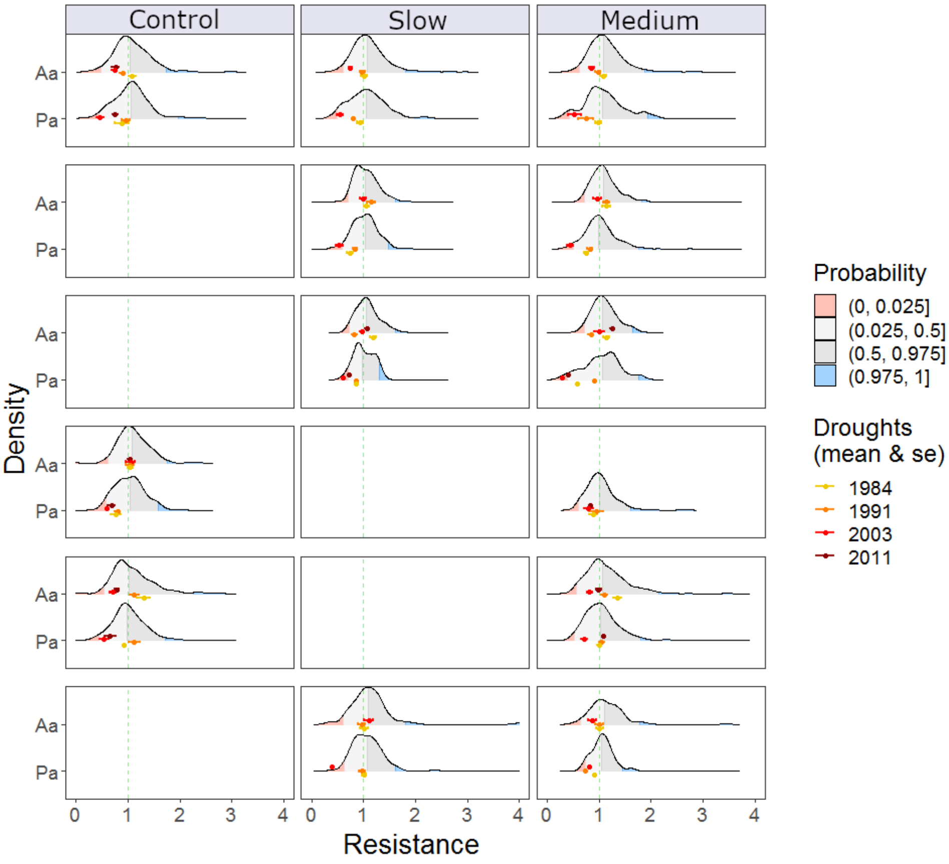
Control
Slow
Medium
Aa
Pa
Density
0
1
2
3
4
Resistance
Probability
(0, 0.025]
(0.025, 0.5]
(0.5, 0.975]
(0.975, 1]
Droughts
(mean & se)
1984
1991
2003
2011

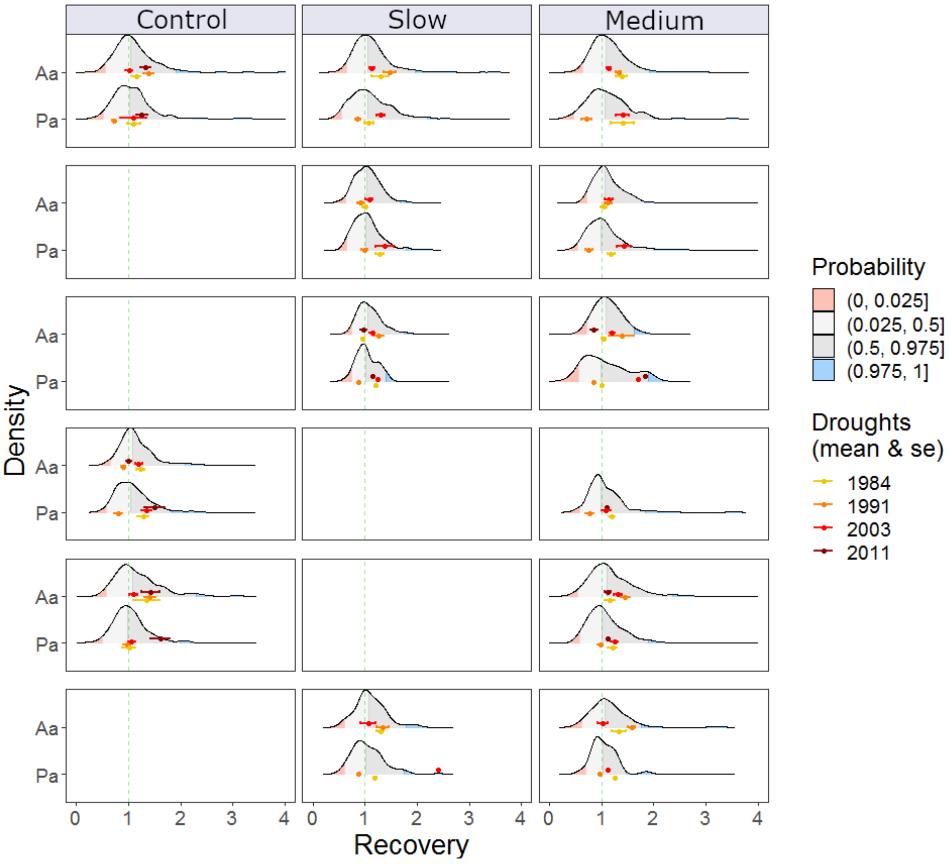
Control
Slow
Medium
Aa
Pa
Density
0
1
2
3
4
Recovery
Probability
(0, 0.025]
(0.025, 0.5]
(0.5, 0.975]
(0.975, 1]
Droughts
(mean & se)
1984
1991
2003
2011

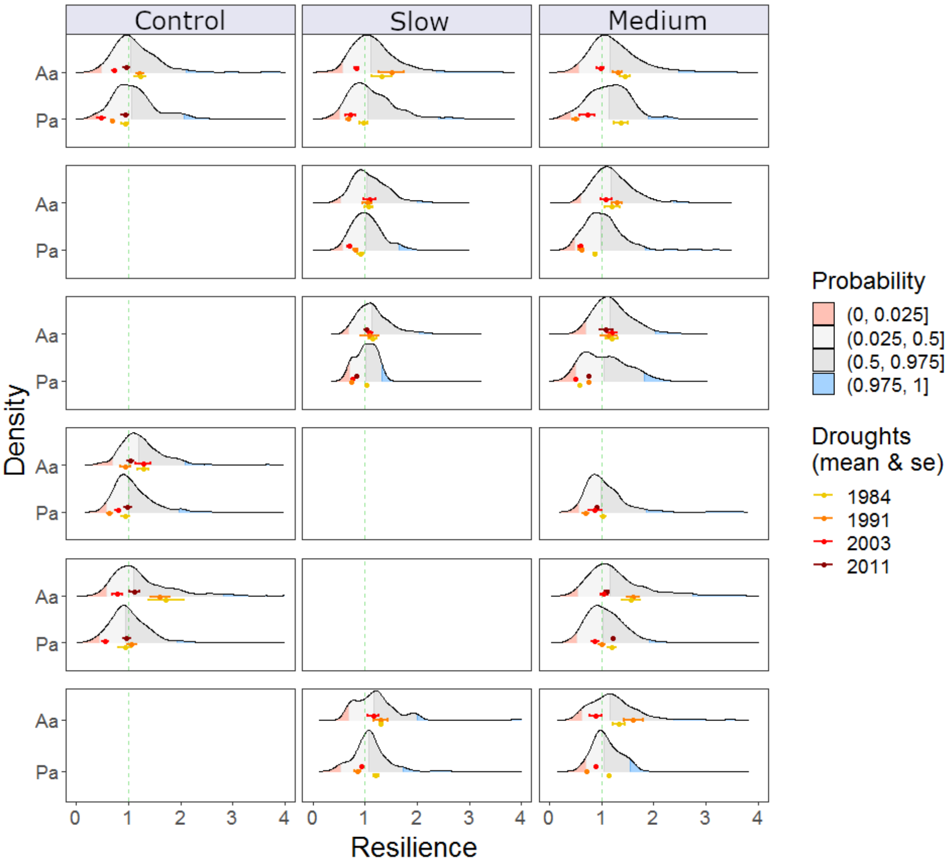

Figure S4: Smoothed density estimates of tree-level drought resistance, resilience and recovery across sites, treatments and species (Aa = *Abies alba*, fir; Pa = *Picea abies*, spruce) for all years in the period 1980-2016. Mean and standard error (se) are reported for the years 1984, 1991, 2003, 2011. The different filling colors represent the probability associated to the density distribution (< 2.5%, 2.5-50%, 50-97.5%, > 97.5%).

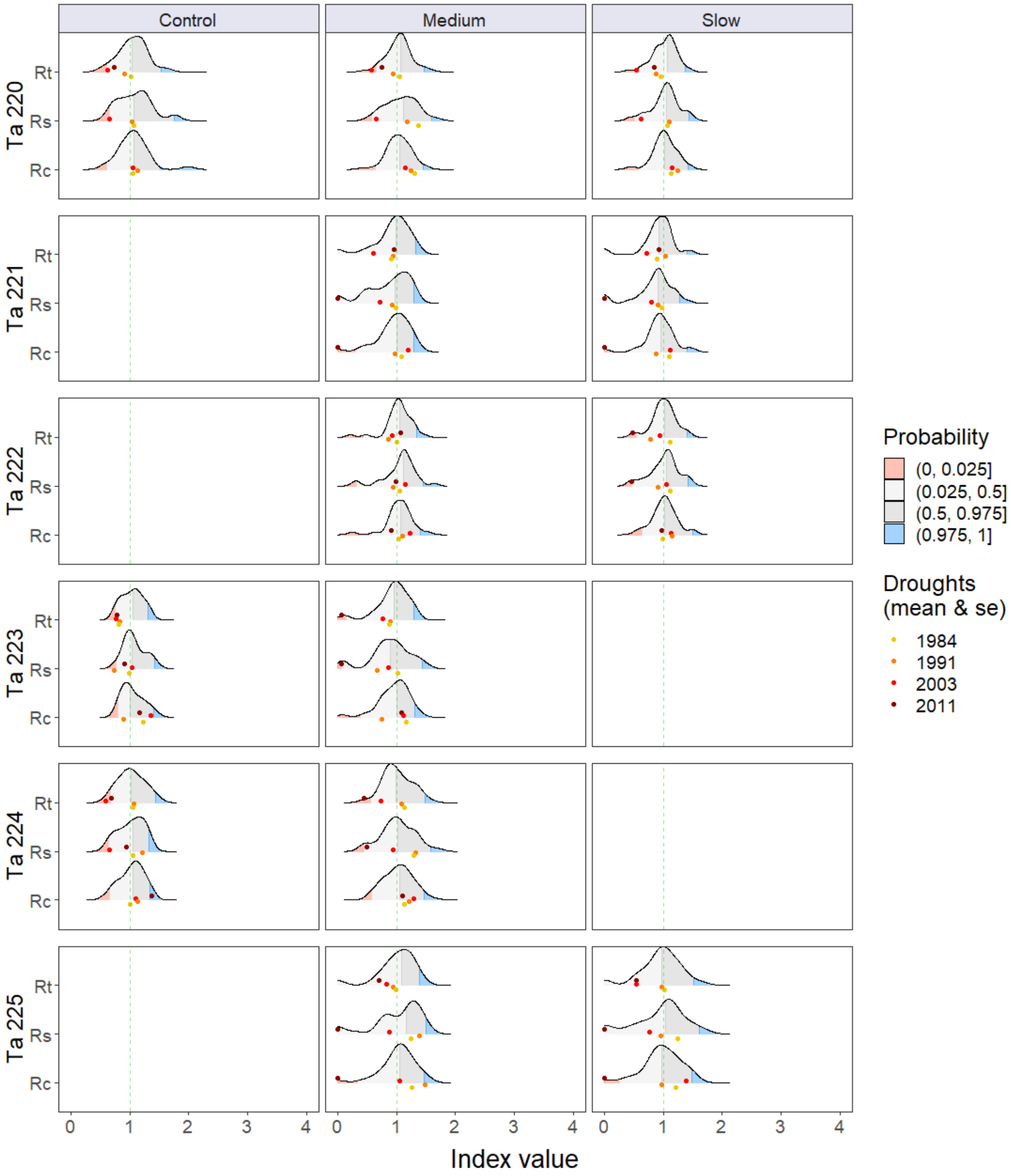

Figure S5: Smoothed density estimates of stand-level drought resistance (Rt), resilience (Rs) and recovery (Rc) across sites and treatments for all years in the period 1980-2016. The years 1984, 1991, 2003, 2011 are highlighted in yellow, orange, red and dark red, respectively. The different filling colors represent the probability associated to the density distribution (< 2.5%, 2.5-50%, 50-97.5%, > 97.5%).

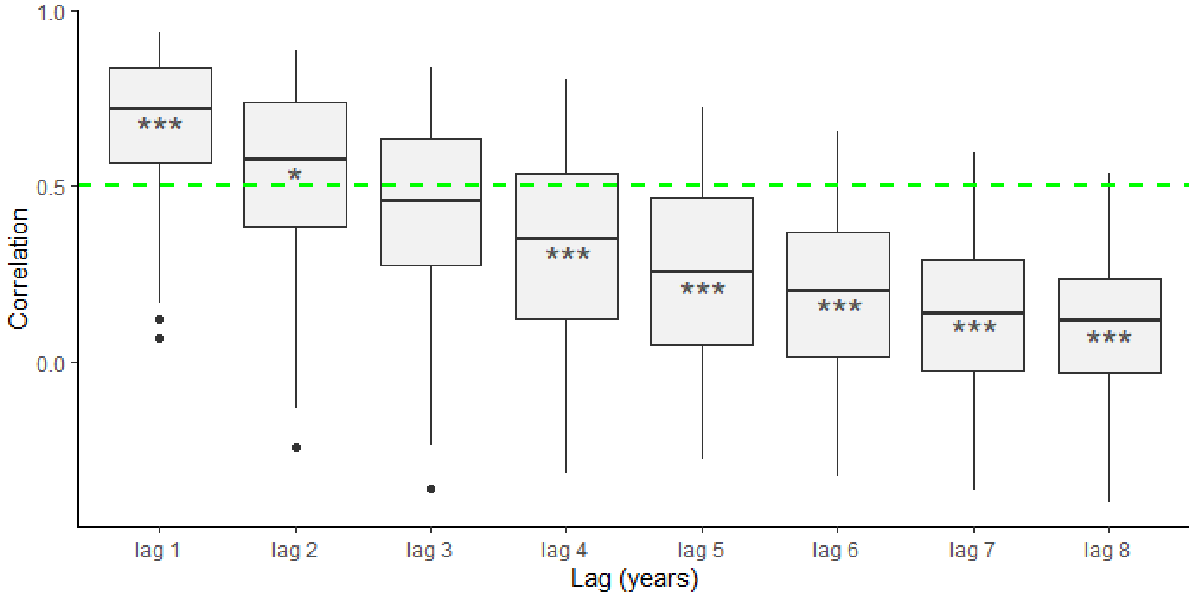

Figure S6: Radial growth autocorrelation for the period 1970-2016. Significance codes for the difference between lag and correlation threshold (0.5; from ANOVA test with post-hoc Tukey Honest Significant Differences): '***' 0.001, '**' 0.01, '*' 0.05, '°' 0.1.

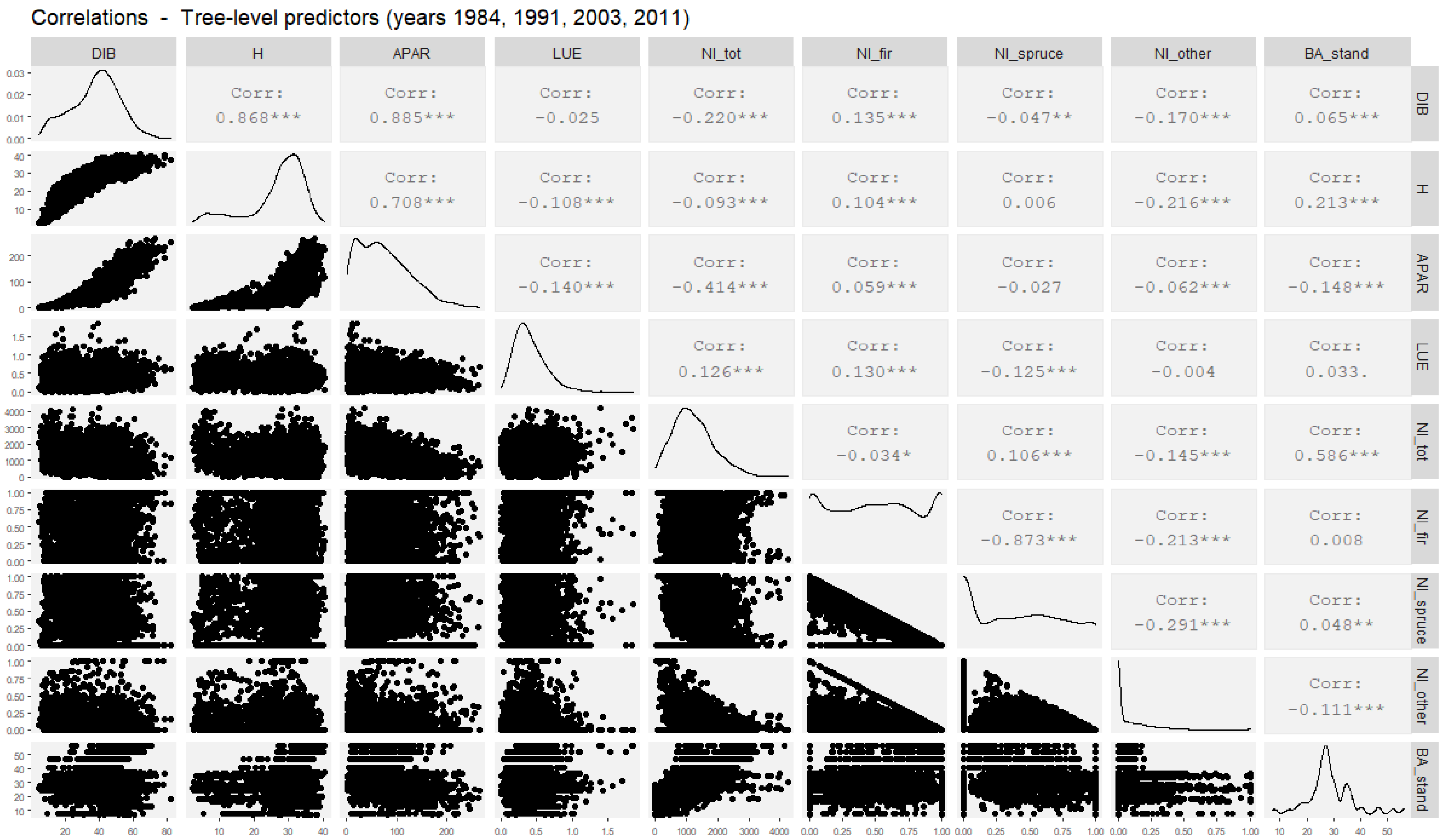

Figure S7: Scatter plot of tree-level variables with correlation coefficients. DIB = diameter inside bark at breast height (cm, measured at 1.3 m height); H = total tree height (m); APAR = absorption of photosynthetically active radiation (GJ/tree/year); NI_tot = competition index (cm^2/m); NI_fir = NI fir (%); NI_spruce = NI spruce (%); NI_other = NI other species (%); BA_stand = total residual stand basal area (m^2/ha).

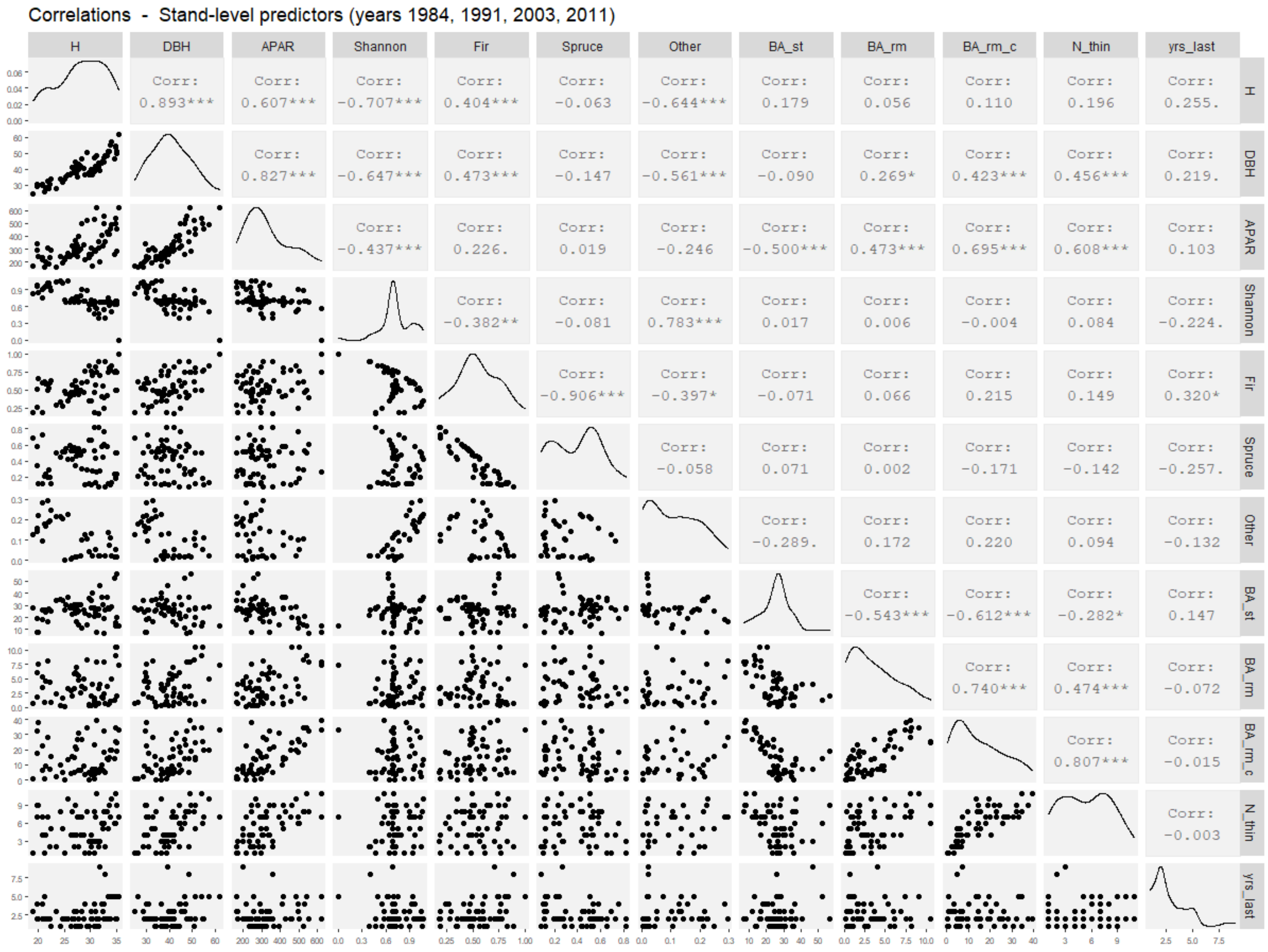

Figure S8: Scatter plot of stand-level variables with correlation coefficients. H = mean stand height (m); DBH = mean stand DBH (cm); APAR = absorption of photosynthetically active radiation (GJ/stand/year); Shannon = Shannon diversity index; Fir = ratio of basal area of fir to stand basal area (%); Spruce = ratio of basal area of spruce to stand basal area (%); Other = ratio of basal area of other species to stand basal area (%); BA_st = total residual stand basal area (m^2/ha); BA_rm = stand basal area removed (m^2/ha); BA_rm_c = cumulative sum of stand basal area removed (m^2/ha); N_thin = total number of interventions; yrs_last = number of years since last intervention.

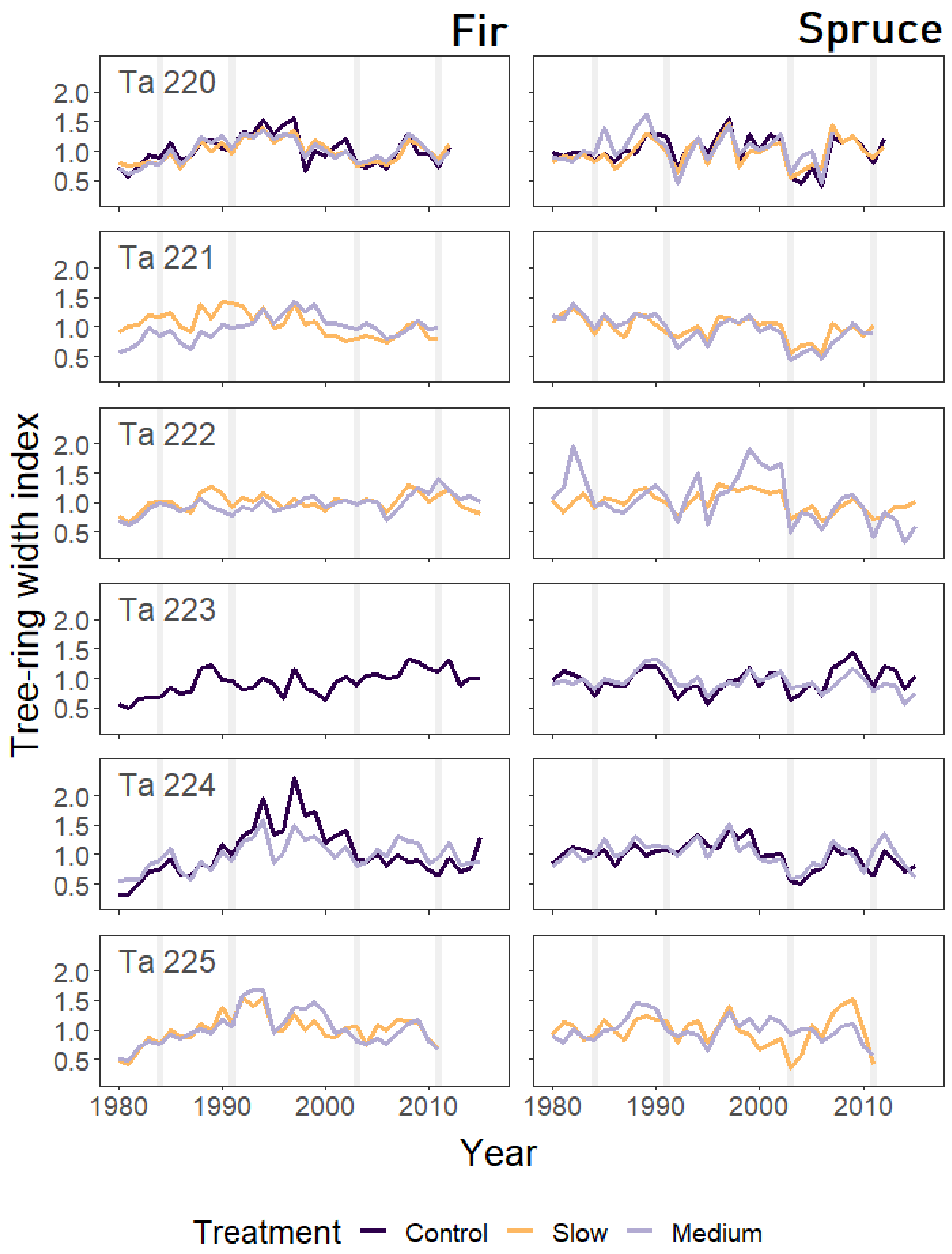

Figure S9. Mean-value site chronology (tree-ring width indices) of fir and spruce across the analyzed treatments and sites since 1980. Vertical grey lines denote the years 1984, 1991, 2003, and 2011. Individual tree-ring series were detrended by a smoothing spline, with 50% frequency response at 2/3 of series' length. Site chronologies were built using the Tukey's biweight robust mean with the function *tbrm* of the R package *dplR* (Bunn et al., 2014).

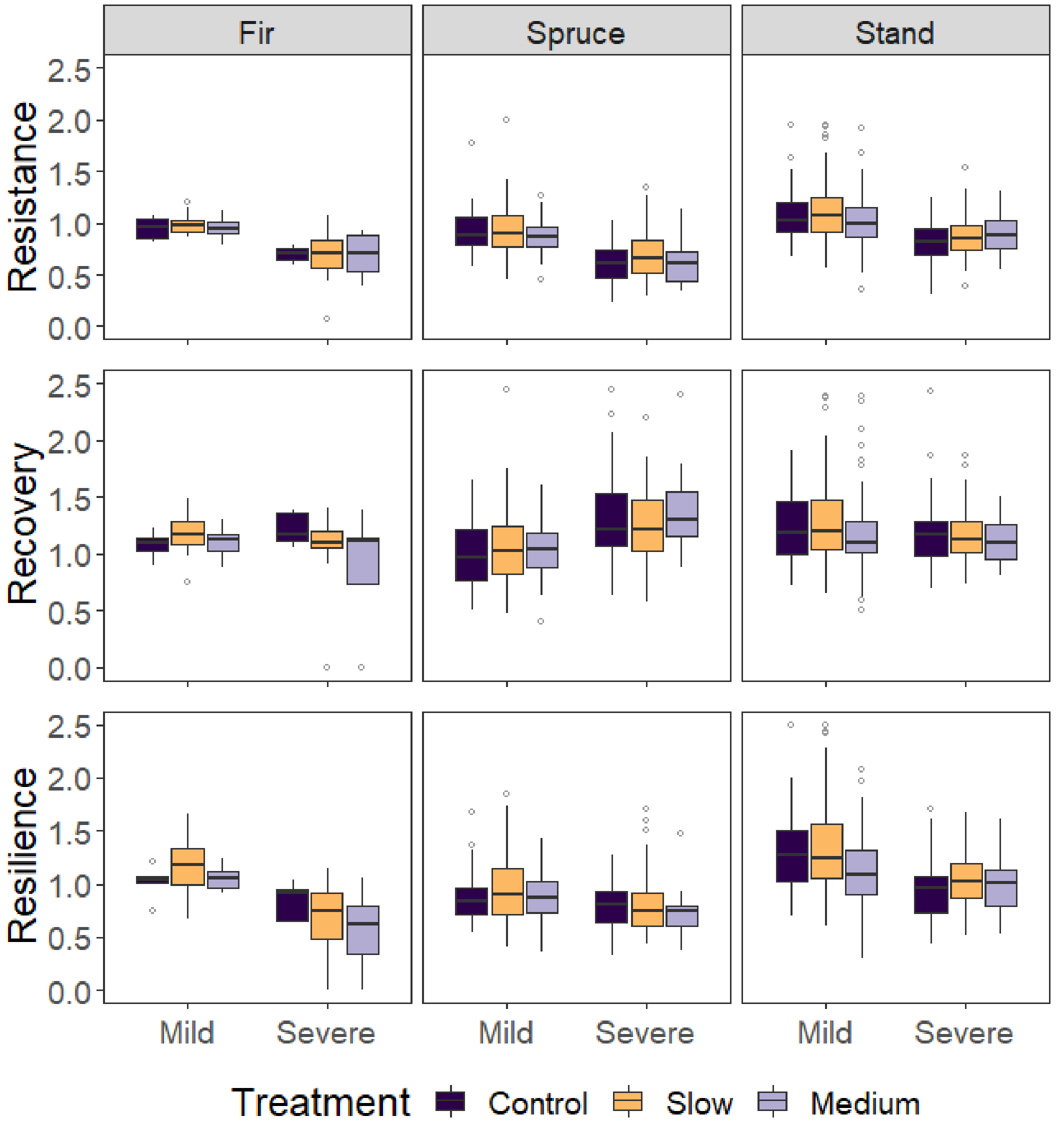

Figure S10. Boxplots of resistance, recovery and resilience by forest component (individual fir, individual spruce, whole stand), drought event (mild, severe) and treatment (control, slow, medium).

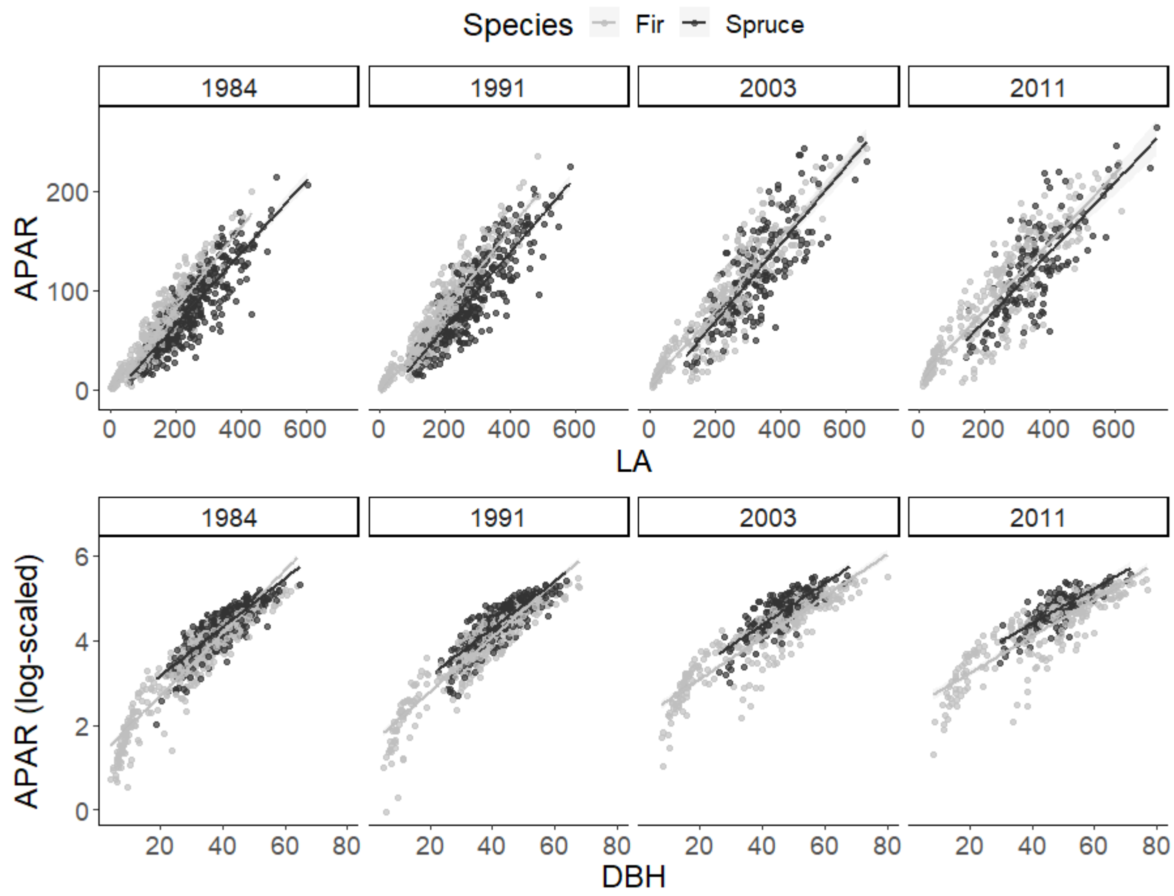

Figure S11: Relationships between APAR and tree characteristics for drought 1984, 1991, 2003 and 2011. APAR = absorption of photosynthetically active radiation; LA = leaf area; DBH = diameter inside bark at breast height.
